# Model-driven exploration of lactose and galactose metabolism via an oxidoreductive pathway in *Sungouiella intermedia* for cell factory applications

**DOI:** 10.1186/s12934-026-02941-y

**Published:** 2026-02-01

**Authors:** Kameshwara V. R. Peri, Iván Domenzain, Hanna D. Alalam, Luca Torello Pianale, Abril Valverde Rascón, Jens Nielsen, Cecilia Geijer

**Affiliations:** 1 Department of Life Sciences, Chalmers University of Technology, Gothenburg, Sweden; 2 BioInnovation Institute, Ole Maaløes Vej 3, DK2200, Copenhagen, Denmark

**Keywords:** Non-conventional yeast, Precision fermentation, Leloir pathway, Galactitol, Tagatose, Flux balance analysis, Genome-scale metabolic model, Candida intermedia

## Abstract

**Background:**

Converting industrial side streams into value-added chemicals using microbial cell factories is of increasing interest, as such processes offer sustainable solutions to reduce waste and production costs. However, developing new, efficient non-model cell factories for precision fermentation remains challenging due to limited knowledge about their metabolic capabilities.

**Results:**

Here, we investigate the lactose and galactose metabolism of the understudied yeast *Sungouiella intermedia* (formerly *Candida intermedia*), using knowledge-matching of high-quality genome-scale metabolic model (GEM) with extensive experimental analysis, and determine its potential as a future cell factory on lactose-rich industrial side-streams. We show that this yeast possesses the conserved Leloir pathway as well as an oxidoreductive route for galactose catabolism. Model simulations and experimental data from continuous and batch bioreactors, transcriptomics, and metabolite analysis indicate that while the Leloir pathway dominates galactose metabolism in *S. intermedia*, the oxidoreductive pathway is employed in a condition-dependent manner. The yeast produces galactitol as a carbon overflow metabolite, facilitating redox cofactor balance during both lactose and galactose growth. Furthermore, the new metabolic insights facilitated the development of an improved bioprocess design, where an engineered *S. intermedia* strain could achieve galactitol yields of > 90% of the theoretical maximum using the industrial side-stream cheese whey permeate as feedstock. Additional strain engineering resulted in galactitol-to-tagatose conversion, proving the versatility of the future production host.

**Conclusions:**

Overall, this work sheds new light on the intrinsic interplay between parallel metabolic pathways that shape the lactose and galactose catabolism in *S. intermedia*. It also demonstrates how a GEM combined with experimental analysis can work in synergy to fast-forward metabolic characterization and development of new, non-model yeast cell factories.

**Supplementary Information:**

The online version contains supplementary material available at 10.1186/s12934-026-02941-y.

## Introduction

Climate change, supply chain instability, and biodiversity loss are intensifying pressures on both society and the environment, underscoring the need to transition toward more resilient and sustainable production systems. In this context, microbial fermentation offers flexible, robust, and scalable solutions, with recent technological advances further expanding its potential [1–3]. Microorganisms such as bacteria, yeasts and fungi can be used as “cell factories” for production of various metabolites of substantial societal and monetary value, including both bulk and speciality compounds. In addition to the well-known baker’s yeast *Saccharomyces cerevisia*e, widely used for production of various pharmaceuticals [4–7] and platform chemicals [8–11], several non-model yeasts have also found applications due to their unique and desirable properties, e.g. *Yarrowia lipolytica* for lipids [4], *Pichia pastoris* for recombinant proteins [5, 6] and *Kluyveromyces lactis* for lactase production [7]. However, there is potential to explore biotechnologically useful functionalities in many more non-model yeasts, thereby expanding the range of chemicals that can be produced efficiently through precision fermentation.

**Fig. 1:**
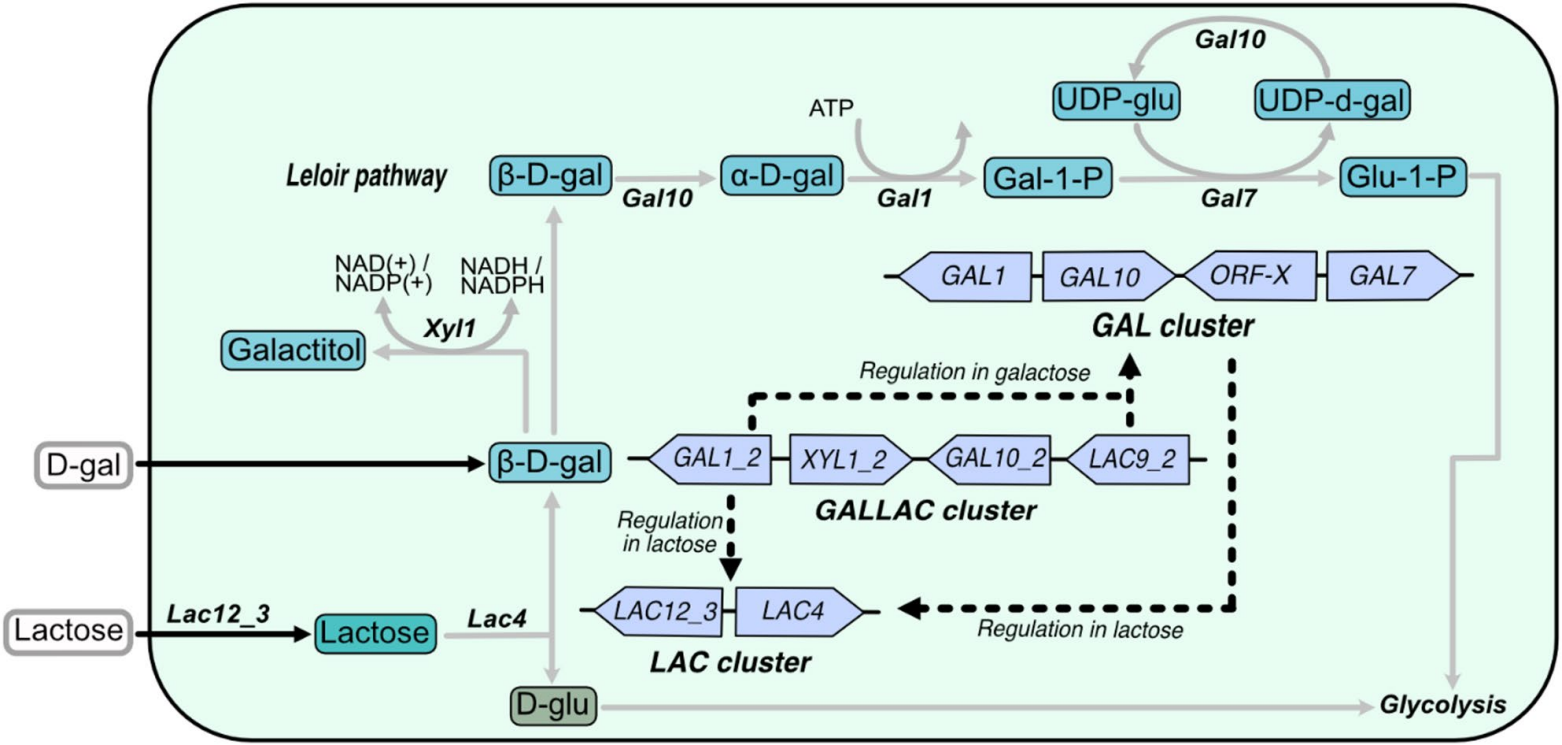
Depiction of the role of gene clusters in lactose and galactose metabolism in *S. intermedia*, adapted from [17]. Lactose metabolism starts with the uptake of lactose and subsequent hydrolysis to β-D-galactose. The *GALLAC* cluster gene *GAL1_2* exerts transcriptional regulation on *LAC* cluster genes (encoding uptake and hydrolysis of lactose) on lactose. *GAL1_2* along with *LAC9_2*, directly or indirectly, regulates *GAL* cluster (Leloir pathway enzyme-encoding genes) expression on galactose. The *GAL* cluster has a regulatory control on the *LAC* cluster on lactose. Black solid rectangle depicts cell membrane. Transport is depicted by black solid arrows with the name of the associated transporter protein above it. Regulation is depicted by black dotted lines. Enzymatic conversion is shown by grey arrows and associated protein above or below. Abbreviations of metabolites are as follows – β-D-galactose (D-gal or β-D-gal); α-D-Galactose (α-D-gal); Galactose-1-phosphate (Gal-1-P); Glucose-1-Phosphate (Glu-1-P); UDP-glucose (UDP-gluc); UDP-galactose (UDP-gal) and D-glucose (D-glu)

The non-model yeast *Sungouiella intermedia*, formerly known as *Candida intermedia* [8], belongs to the CUG-Ser1 clade of ascomycetous yeasts and has been studied for its ability to assimilate xylose and convert lignocellulosic hydrolysates into valuable products [9–15]. Recent development of genome editing tools for this yeast [16] has facilitated investigation also of its lactose and galactose metabolism, revealing novel regulatory, transcriptional and catabolic mechanisms involving three distinct gene clusters: the *LAC* cluster (*LAC12* lactose permease and *LAC4 β*-galactosidase), the *GAL* cluster (*GAL1*, *GAL10* and *GAL7* for galactose catabolism via the Leloir pathway), and a unique *GALLAC* cluster (second copies of *GAL1_2* and *GAL10_2*, transcriptional regulator *LAC9_2* and an aldose reductase *XYL1_2*) [17] (summarized in Fig. 1). Phenotypic analysis of gene deletion mutants revealed that the *GALLAC* cluster is indispensable for growth on lactose or galactose and signified the roles of Lac9 and Gal1_2 as key metabolic regulators. Overall, this work revealed a complex interdependence between the gene products from the three clusters, where many of the proteins seem to exert both regulatory and enzymatic functions.

Interestingly, we observed that deletion of the conserved *GAL* cluster in *S. intermedia*, which disrupts the Leloir pathway for galactose metabolism, resulted in slow growth on lactose along with production and extracellular accumulation of galactitol [17], a sugar alcohol with applications in the food industry as a precursor of the natural sweetener tagatose [18, 19]. Current industrial production of galactitol relies mainly on the chemical hydrogenation of galactose to galactitol, a process plagued by high costs and inefficiencies [20]. Moreover, fermentation-based production of tagatose is an active area of research, with various microbial platforms being explored [20–22]. In this context, *S. intermedia*’s ability to convert lactose into galactitol offers a new route to develop whey-based processes using this abundant, lactose-rich dairy side stream.

Engineering a new production host that can reach high titers, yields and productivities in the bioproduction process, requires extensive understanding of its overall metabolism [23]. This can be achieved through a combination of experimental characterization and a systems level approach using a genome-scale metabolic model (GEM), which is a structured knowledge base of a cell’s metabolism in the form of a collection of (i) genes in the genome, (ii) metabolites in the cell and (iii) reactions that they partake in [24]. In GEMs, laws of thermodynamics and biochemical stoichiometry define and constrain the relations between such components, and thus, the cell itself [25, 26], enabling quantitative simulations of metabolism under genetic and environmental perturbations and linking genotypes to phenotypes [27, 28]. They can also facilitate a detailed understanding of a cells’ ability to convert substrates into products [29, 30] and are being extensively used to drive metabolic engineering projects in different application areas such as food [31], pharma [32–34] and human health [35–37].

With the overarching aim of understanding the complex lactose and galactose metabolism in *S. intermedia*, we curated and standardized a pre-existing model to generate a high-quality GEM, referred to as *Sint-GEM*. Combined with experimental results, we created a platform for probing the organism’s growth capabilities on lactose and galactose. Furthermore, we explored the potential of *S. intermedia* as a future cell factory for production of galactitol and its derivative tagatose; two metabolites with interesting characteristics for the food and pharma industries.

## Results and discussion

### *Sungouiella intermedia* can catabolize and grow on galactitol

**Fig. 2:**
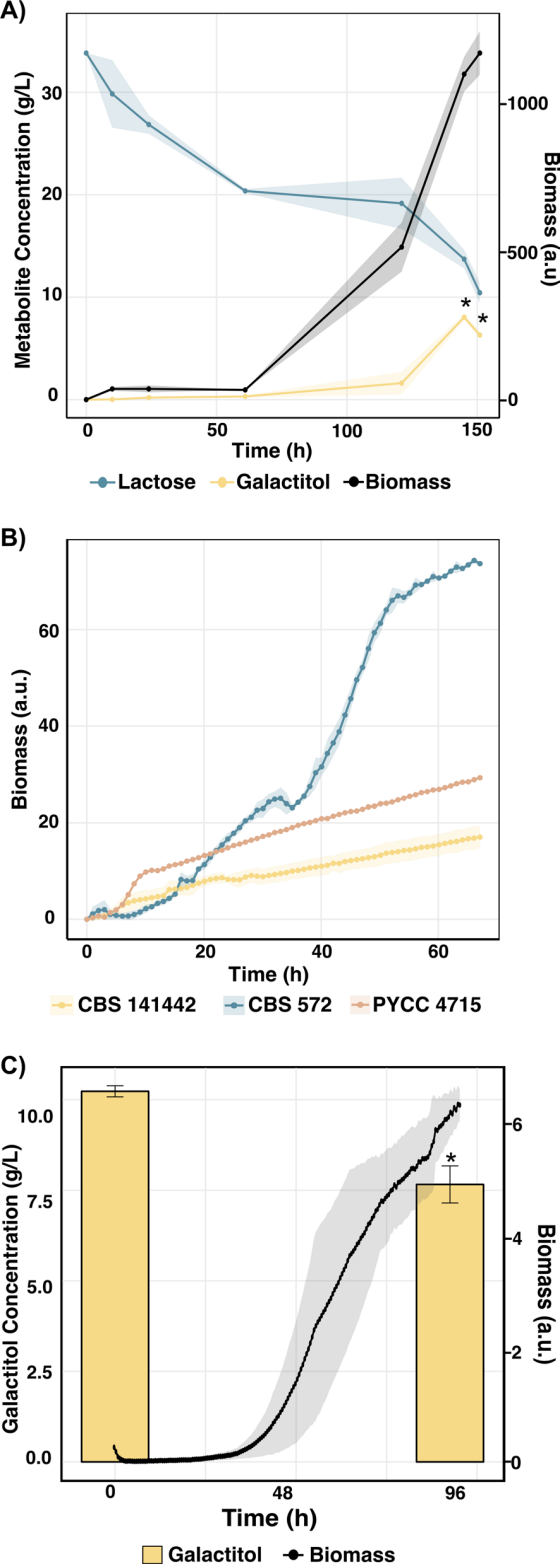
Galactitol production, consumption, and growth in *Sungouiella intermedia*. **A** Growth curve of the *galΔ* mutant grown in minimal media containing lactose as carbon source in shake flasks. Biomass (a.u.) (black line) is plotted on the right y-axis and metabolites (lactose and galactose concentrations in g/L) are plotted on the left y-axis: lactose (blue) and galactitol (yellow) against Time (in hours) are plotted on the x-axis. Data are represented for biological duplicates with variation in growth depicted by shaded region for biomass and error bars for metabolites. Statistical significance is determined using student’s t-test and is represented as “*” for *P* ≤ .05, “**” for *P* ≤ .01, and “***” for *P* ≤ .001. **B** Growth curves of three *S. intermedia* strains (CBS 572 (blue); PYCC 4715 (orange); CBS 141442 (yellow)) on minimal media with 1% galactitol as carbon source. Strains were precultured in lactose before starting the main culture in the Growth Profiler960 (Enzyscreen, The Netherlands). Biomass (a.u.) plotted on y-axis against time (in hours) on x-axis. Data are represented for biological triplicates with variation depicted by shaded region for biomass. **C** Growth profile for *galΔ* mutant cultured in minimal media containing 1% galactitol in micro-titer plate format in the Growth Profiler960 (Enzyscreen, The Netherlands). Biomass (a.u.) is plotted on right y-axis and galactitol concentration (g/L) depicted by bar plot on left y-axis, against Time (in hours) on x-axis. Data are represented for biological triplicates with variation depicted by shaded region for biomass and error bars for galactitol concentration. Statistical significance is determined using student’s t-test and is represented as “*” for *P* ≤ .05, “**” for *P* ≤ .01, and “***” for *P* ≤ .001

As noted above, our earlier work showed that deleting the conserved *GAL* cluster in the *S. intermedia* strain CBS 141442 led to galactitol production during growth on lactose [17]. When we repeated the growth experiment and extended the cultivation time to 150 h, we observed that the *galΔ* strain was not only capable of producing galactitol, but it could also consume it (Fig. 2A). Several filamentous fungi, including *Trichoderma reesei* and *Aspergillus niger*, metabolize galactose through an oxidoreductive pathway involving galactitol [38, 39], and recent work shows that the *S. intermedia* type strain CBS 572 is among the 96 (out of 830) yeast and yeast-like species that can grow on galactitol [40]. Growth of three *S. intermedia* strains, the type strain CBS 572, our lab strain CBS 141442 and PYCC 4715 [41, 42], in minimal media with galactitol as the sole carbon source confirmed that this is a general trait for *S. intermedia*, although the strain CBS 572 grew faster and reached higher OD levels than the other two strains within the time frame of the experiment (Fig. 2B). To determine whether galactitol is metabolized via an oxidoreductive pathway, rather than being first converted to galactose and then processed through the Leloir pathway, we also grew the *galΔ* strain on galactitol as the sole carbon source. The mutant grew and actively consumed galactitol (Fig. 2C), demonstrating that *S. intermedia* possesses a galactitol utilization pathway operating in parallel to the Leloir pathway.

### *Sint-GEM* predicts metabolites belonging to an oxidoreductive pathway

**Fig. 3:**
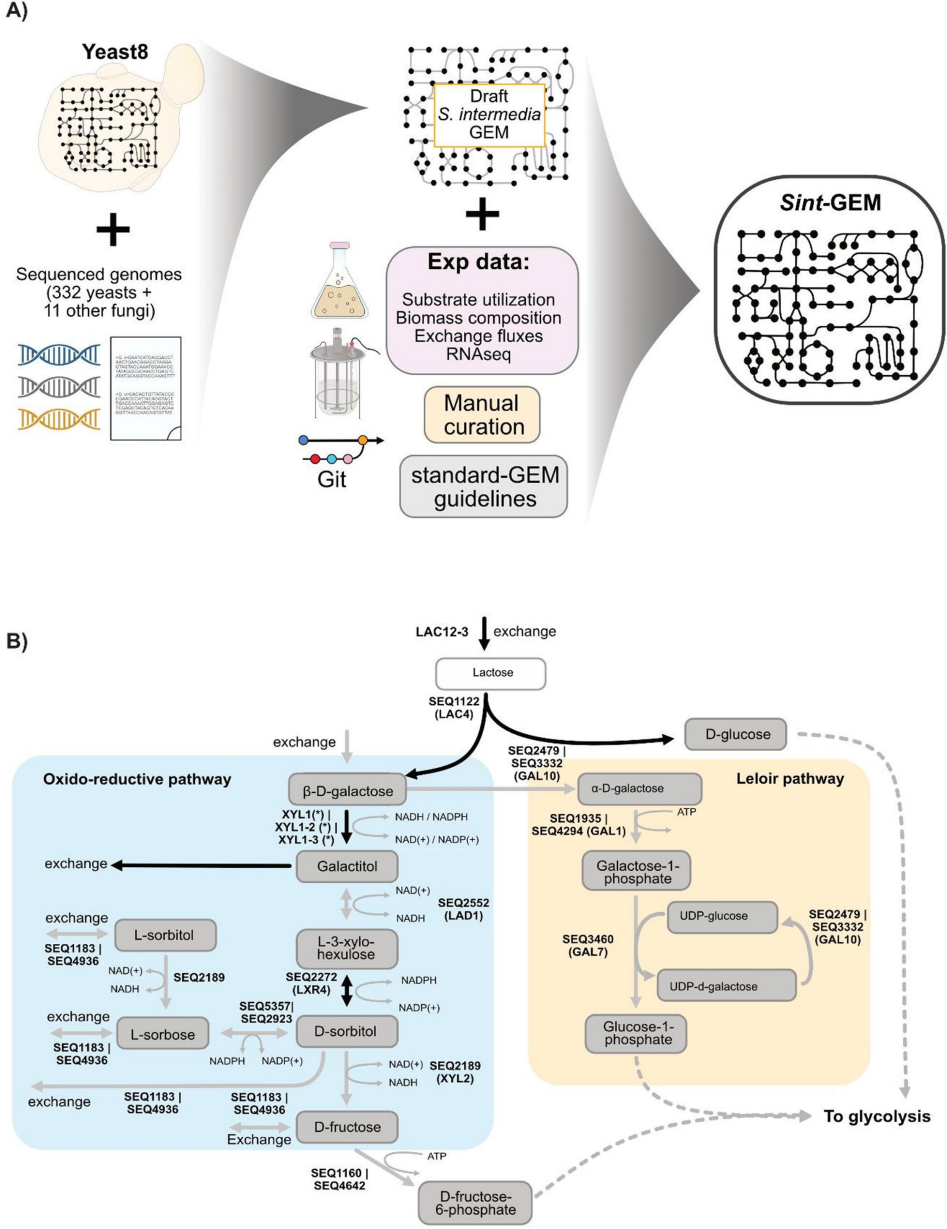
Reconstruction of a GEM for *S. intermedia*. **A** Reconstruction of the *Sint-GEM* using draft model for *S. intermedia* based on yeast8 and gene orthology predictions. Data-driven manual curation and git-based version control yielded a high-quality GEM adhering to standard modeling practices. *Sint-GEM* consists of 1070 genes, 3991 reactions, 2759 metabolites and 14 cellular compartments. **B** Representation of lactose and galactose metabolism in *Sint-GEM*. Curation of the oxidoreductive pathway in *Sint-GEM* involved addition of L-xylo-3-hexulose reductase reaction connecting L-xylo-3-hexulose with D-sorbitol (indicated with black arrows). Additionally, cofactor usage of galactose reductase was incorporated into the model according to experimental results

To investigate the oxidoreductive pathway in *S. intermedia* and the potential benefits of having two parallel galactose catabolic routes, we developed a GEM, hereafter referred to as *Sint-GEM*. A draft model had previously been generated [43] using the *S. cerevisiae* Yeast8 consensus GEM [44] and orthology across 332 yeasts and 11 fungi (Fig. 3A).

An initial assessment of the draft model revealed a missing lactose uptake step, absent in *S. cerevisiae* but supported by experimental evidence in *S. intermedia* [17], which we therefore incorporated into the model (Fig. 3B). In contrast, it already included all metabolites and enzymes associated with the conserved Leloir pathway, as well as the galactose-to-galactitol conversion step catalyzed by an aldose reductase. *S. intermedia* possesses no less than three paralogous genes encoding aldose reductases, previously identified as xylose reductases [14], including Xyl1_2 that is encoded from the *GALLAC* cluster [17]. All three enzymes and their cofactor specificities were incorporated in the model, where Xyl1_2 displays dual redox (NADH/NADPH) cofactor specificity (Fig. S1), while Xyl1_1 and Xyl1_3 are strictly NADPH dependent [14]. As extracellular galactitol accumulation and subsequent consumption were observed in the *galΔ* mutant strain (Fig. 2A), an exchange reaction for galactitol transport/diffusion into and out of the cell was also integrated into the model.

Notably, the model already included the reaction steps for converting galactitol to L-xylo-3-hexulose, predicted to be catalyzed by *LAD1*-encoded galactitol dehydrogenase, as well as the conversion of D-sorbitol to D-fructose by the *XYL2*-encoded D-sorbitol dehydrogenase (Fig. 3B). To complete the oxidoreductive pathway, the reaction converting L-xylo-3-hexulose to D-sorbitol by L-xylo-3-hexulose reductase was added. Here, the *LXR4* gene product was assigned this enzymatic activity, based on ortholog predictions between *S. intermedia* and filamentous fungi possessing an oxidoreductive pathway for galactose catabolism [45]. Together, these reaction steps connect all metabolites from β-D-galactose to D-sorbitol, and further to D-fructose-6-phosphate for glycolytic assimilation. In addition, *Sint-GEM* contained additional reactions involving possible oxidoreductive pathway intermediates, including the conversion of D-sorbitol to L-sorbose by Seq_5357 or Seq_2923 and L-sorbitol to L-sorbose by Seq_2189. Table S1 summarizes the rationale behind our assumptions regarding the structure of the oxidoreductive pathway.

To further refine the predictive capabilities of *Sint-GEM*, data on biomass composition and consumption/secretion rates of metabolites from continuous cultivations were incorporated and utilized to fit the model’s energy requirements. The development of *Sint-GEM* was tracked following standard-GEM guidelines [46] and can be found at: https://github.com/SysBioChalmers/sint-GEM.

### Transcriptional insights support the *Sint-GEM*-predicted oxidoreductive pathway

**Fig. 4:**
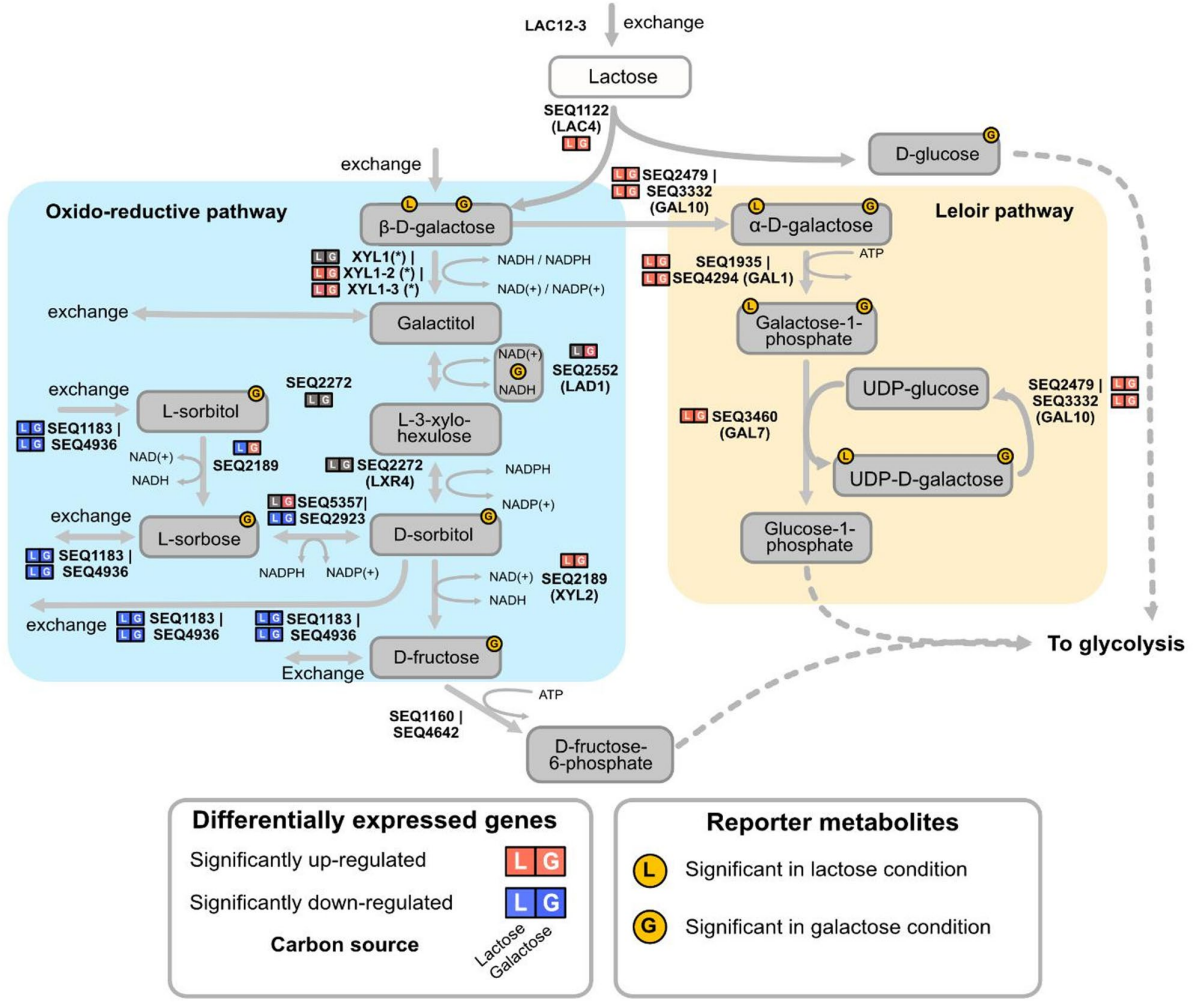
Transcriptional insights support activity of the *Sint-GEM*-predicted oxidoreductive pathway during growth on lactose and galactose. Differential expression profiles and reporter metabolite analysis of genes and pathway intermediates of the Leloir pathway and the hypothesized oxidoreductive pathway in *S. intermedia* wild-type strain on galactose and lactose. Differential expression of genes is depicted by squares indicating directionality (red = upregulation; blue = downregulation) and the utilized carbon source (indicated by L = lactose and G = galactose). Similarly, reporter metabolites are represented by yellow circles indicating the carbon source in which the metabolite is being characterized

Attempting to elucidate the network topology of the oxidoreductive pathway, we started by contextualizing the differential gene expression observed during galactose and lactose growth compared to glucose growth (reported previously in [17]), in *Sint-GEM*. An overview of the transcriptional profiles for the predicted pathway genes is shown in Fig. 4. Metabolic contextualization of the transcriptional data depicted a cellular condition in which the Leloir pathway enzymes are highly expressed. Moreover, expression of the aldose reductases Xyl1_2 and Xyl1_3 suggests that the encoded enzymes pull flux toward conversion of galactose into galactitol. Galactitol can be secreted out of the cell, as observed in the *galΔ* strain, or further catabolized by expressed enzymes such as Lad1 and Xyl2, to provide additional glycolytic flux. Additionally, strong downregulation of genes encoding for transporters of other oxidoreductive metabolites (L-sorbose and D-sorbitol) can minimize the loss of additional carbon flux. Overall, based on gene expression profiles, flux through both the Leloir and the oxidoreductive pathway in *Sint-GEM* suggests a possible coordinated effort in maximizing carbon assimilation.

Additionally, we performed a reporter metabolite analysis [47], using the differential gene expression data for cells grown on lactose or galactose relative to glucose [14]. Results for both carbon sources showed that four out of the six metabolites participating in the Leloir pathway become significant nodes of transcriptional regulation (adjusted p-value < 0.001), i.e. indicating strong and coordinated transcriptional up/down-regulation of genes associated with these metabolites (Fig. 4). In contrast, among the predicted metabolites in the oxidoreductive pathway, only β-D-galactose was found among the top reporter metabolites on both carbon sources (galactitol exhibited a similar positive trend but did not meet the threshold set for significance). Moreover, several other metabolites in the oxidoreductive pathway (such as NAD(+/H), D-sorbitol, L-sorbose and L-sorbitol) were found to be nodes of significant regulation only on galactose. These results suggest that lactose availability preferentially activates the Leloir pathway for metabolizing the galactose moiety, whereas growth on galactose as the sole carbon source appears to induce both the Leloir and oxidoreductive pathways. Overall, this points towards a transcriptional machinery that supports a role of the hypothesized oxidoreductive pathway in galactose metabolism in *S. intermedia*.

### Deletion mutant phenotyping suggests enzyme promiscuity

The network topology of *Sint-GEM*, featuring the hypothesized oxidoreductive pathway, prompted the construction of deletion mutants to verify the involvement and function of specific enzymes within this pathway. To this end, we assessed growth on galactitol for wild-type and the *lad1Δ*,* lxr4Δ* and *xyl2Δ* mutants, where the deleted genes normally encode enzymes predicted by Sint-GEM to act downstream of galactitol in the oxidoreductive pathway (Table S1). All three mutants could grow on galactitol. In fact, *lad1Δ* and *xyl2Δ* grew somewhat faster than wild-type and *lxr4Δ*, although all strains reached comparable final optical densities (Fig. S2). The lack of distinct growth defects on galactitol may be a consequence of a high degree of substrate promiscuity of oxidoreductive enzymes, as demonstrated previously in other fungi [38, 39], where multiple gene deletions are often required to reveal clear growth phenotypes. Alternatively, the carbon flux may be routed through other enzymes that are not captured in the *Sint-GEM.* Such pathway flexibility is consistent with the known variation in the architecture of the galactose oxidoreductive pathway among fungi, even though the core principle of conversion of galactose to galactitol followed by oxidoreductase-mediated metabolism is conserved [48].

The failure to identify single mutants unable to grow on galactitol, combined with the difficulties of constructing multiple mutants in the same strain background due to limited genetic tools in *S. intermedia* [16], prompted us to take an alternative approach. We therefore performed a correlation analysis of gene expression levels (RNA-seq) across multiple carbon sources (glucose, lactose, galactose, cellobiose, and xylose) (Fig. S3A-B), to identify genes that are co-regulated and potentially involved in lactose and galactose metabolism. We found that the expression of *XYL1_2* (located in the *GALLAC* cluster) was tightly correlated to expression of the Leloir pathway genes, as well as the lactose transporter *LAC4* gene (p-value ≤ 0.01) [17]. Moreover, a significant positive correlation was observed for *LAD1*, *XYL1_2*, and *XYL1_3* as well as between *LAD1* and all genes in the Leloir pathway. *LXR4* showed significant correlation to *XYL1_2*,* LAD1*,* GAL1_2* and *LAC9*, while *XYL2* was found to be significantly correlated only with *XYL1_2* expression. Together, these results suggest extensive co-expression and possible co-regulation between the Leloir and oxidoreductive pathways as seen in *Sint-GEM*, with evidence of crosstalk between them.

Given these findings, our subsequent *Sint-GEM* analyses focused on capturing the overall metabolic behavior of the Leloir and oxidoreductive routes rather than resolving each individual reaction within the oxidoreductive pathway. Accordingly, we prioritized interpreting model predictions at the level of overall pathway flux and detectable substrates (galactose and lactose) and metabolites (galactitol and ethanol).

### Flux balance analysis suggests conditional use of the oxidoreductive pathway

Next, to evaluate the functionality of the oxidoreductive route during galactose and lactose metabolism in *Sint-GEM*, we performed flux balance analysis (FBA) to predict growth capabilities on each carbon source [49]. In both carbon sources, the simulated flux distributions indicated that the Leloir pathway remains the stoichiometrically optimal route for assimilating the galactose moiety, as its deletion consistently reduced the simulated biomass yield. To avoid confounding effects from the glucose moiety in lactose, we focused on analyzing the flux distributions on galactose as the sole carbon source. Under this condition, forcing assimilation through the oxidoreductive pathway resulted in only a 3.14% decrease in biomass yield. This modest reduction was primarily associated with a 2.27-fold increase in glycolytic flux diversion toward the pentose phosphate pathway (PPP) for NADPH regeneration compared with the optimal Leloir-utilizing phenotype (Suppl. Table 2). These results suggest that although the Leloir pathway is preferred, the oxidoreductive pathway is fully capable of carrying flux and stoichiometrically supporting growth.

To identify the conditions that support flux through the oxidoreductive pathway in *Sint-GEM*, we also performed random flux sampling [50] using galactose or lactose as carbon sources under carbon-, oxygen-, and nitrogen-limited growth. For each condition, 10,000 FBA simulations with randomized objectives were run, generating a diverse set of feasible metabolic phenotypes. These simulations showed that the Leloir pathway carried flux in > 99% of cases across all conditions tested (Suppl. Table 2). Nevertheless, simultaneous use of both pathways was common. For example, for galactose, dual-pathway flux appeared in 100% of oxygen-limited simulations and 42.6% of nitrogen-limited simulations. For lactose, it occurred in 60.9% of oxygen-limited simulations. In contrast, exclusive use of the oxidoreductive pathway occurred in < 1% of all sampled distributions, indicating that its activation is condition-dependent rather than constitutive.

**Fig. 5:**
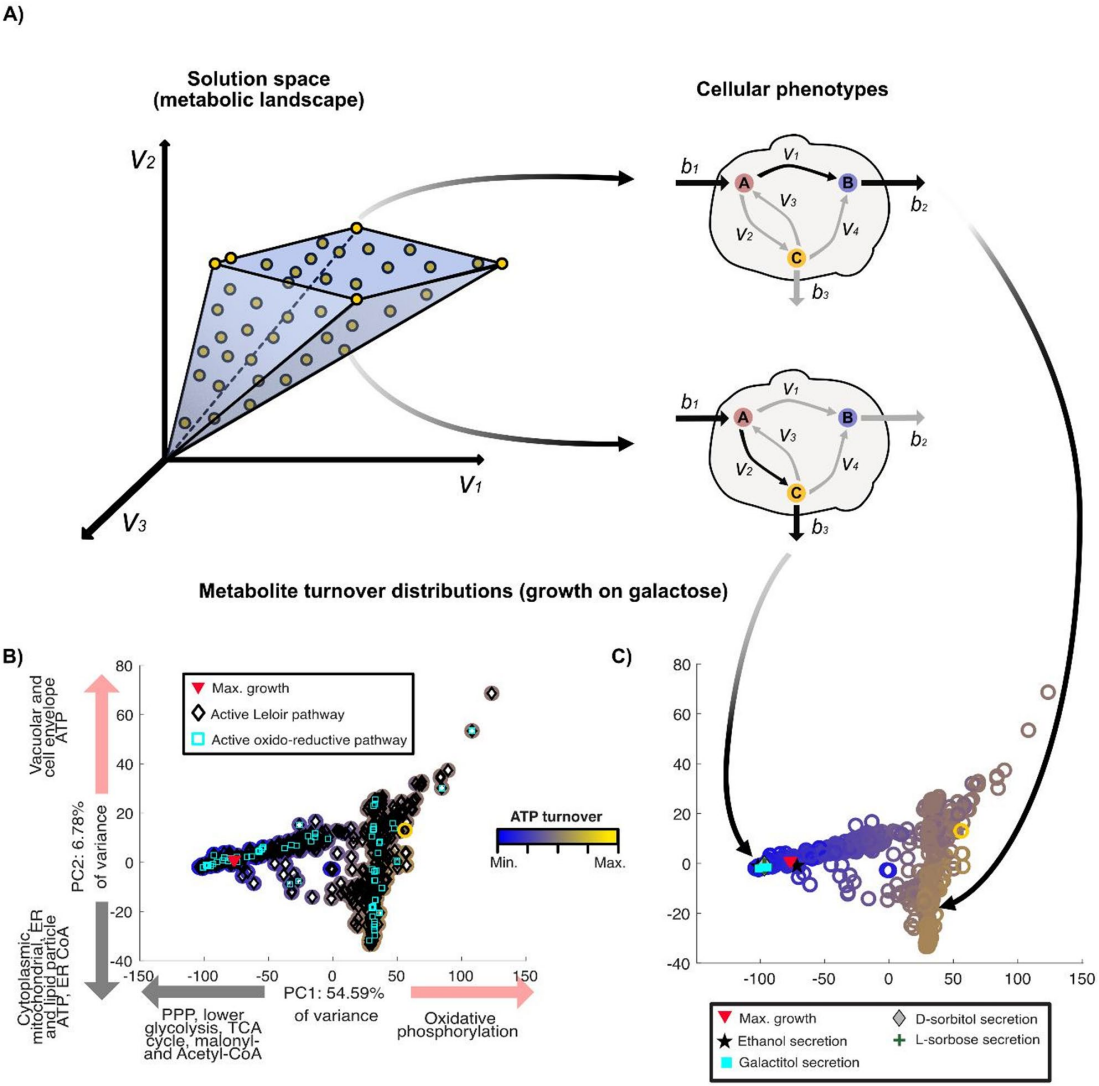
Principal component analysis (PCA) of metabolic flux simulations from *Sint-GEM*, illustrating metabolic turnover profiles of *S. intermedia*: **A** FBA simulations with random objective function are computed, allowing the exploration of attainable cellular phenotypes given a set of specified constraints. **B**, **C** Principal component analysis of flux distributions. Metabolite turnover distributions are computed from flux distributions and dimensionally reduced using principal component analysis. Sampled FBA simulations are mapped into PC1-PC2 space. Arrows and legend next to PCs axis represent the metabolites (also related to pathway or subcellular localization) whose turnover rates are the top contributors to the respective PC in both positive and negative directions. Both (**B**) and (**C**) represent the same 10,000 simulations of cellular growth under carbon-limited conditions with galactose as sole carbon source. Color of each sample (rings) represents the normalized total intracellular ATP turnover (dark blue for minimal values, bright yellow for maximal ones). **B** The use of the Leloir pathway and/or the oxidoreductive pathway for galactose catabolism is mapped onto each of the sampled distributions. Simulations achieving the maximum optimal growth are marked by a red inverted triangle. **C** FBA simulations, where secretion of galactitol, D-sorbitol, L-sorbose and ethanol is mapped into the PC1-PC2 space of the sampled metabolite turnover distributions

Flux distributions from random sampling FBA were transformed into metabolite turnover distributions followed by a dimensionality reduction using principal component analysis (PCA) (see materials and methods for further details), to obtain a global view of the *Sint-GEM* solution space (Fig. 5A, Figure S4A-F). Under both galactose- and lactose-limited conditions, flux distributions through the Leloir and oxidoreductive pathways showed substantial overlap (Fig. 5B), a pattern that also appeared under nitrogen limitation for both carbon sources (Fig. S4 A-F). In contrast, oxygen limitation led to clearer separation between the two pathway usage profiles, likely reflecting the distinct glycolytic contributions during lactose versus galactose metabolism.

Interestingly, random sampling on both carbon sources showed that > 95% of oxidoreductive-pathway flux distributions preferred NADH over NADPH, suggesting that the dual cofactor specificity of *S. intermedia’s* aldose reductase Xyl1_2 serves an important physiological role. This is notable, as dual cofactor specificity is rare among yeast aldose reductases [51, 52]. Moreover, random sampling suggested that exclusive NADPH usage was uncommon, with the only notable increase occurring under lactose-limitation, where NADPH-related activity appeared in ~ 45% of simulations. However, most of these cases reflected NADH/NADPH dual cofactor usage (~ 42%), meaning that truly NADPH-exclusive phenotypes remained rare. Simulations in which the Leloir pathway was blocked, further confirmed NADH as the optimal cofactor for galactitol formation. When both the Leloir pathway and NADH usage were restricted, simulations displayed a strong shift toward PPP-mediated NADPH regeneration, with a 2.8-fold increase in PPP flux and a corresponding reduction in biomass yield (Suppl. Tables 3 and 4). Together, these results underscore the importance of Xyl1_2’s cofactor flexibility, suggesting that NADH usage stabilizes intracellular redox balance through galactitol formation.

### Model simulations and quantitative fermentation demonstrate galactitol secretion under nutrient-limited conditions

As shown experimentally (Fig. 2A), the *S. intermedia galΔ* strain accumulates galactitol extracellularly when grown on lactose. This motivated analysis of secretion fluxes across all simulations explored through random sampling, aiming to identify the conditions under which galactitol and other putative metabolites in the oxidoreductive pathway (D-sorbitol, L-sorbose) might be secreted. We also mapped ethanol, as this is a metabolite that is formed during oxygen-limited conditions in many yeasts, including *S. intermedia* [14]. Simulated secretion profiles of the wild-type strain were overlaid onto the PCA plots (Fig. 5C for galactose), which were generated from randomly sampled metabolite turnover distributions. These analyses were performed under carbon-, nitrogen-, and oxygen-limited conditions for both galactose and lactose (Fig. S5A-F), with the results described in separate sections below.

### Carbon-limiting conditions

Random sampling under carbon-limiting conditions in lactose suggested that only a minor fraction of the simulations displayed secretion of any of the target metabolites (less than 1% for all cases, Table 1, Fig. S5B). Similarly, cultivation of the wild-type strain in a lactose-limited chemostat at two different dilution rates showed no accumulation of the metabolites (data not shown). This suggests that the strain grew under optimal, carbon-limited conditions and relied primarily on respiratory metabolism.

**Fig. 6:**
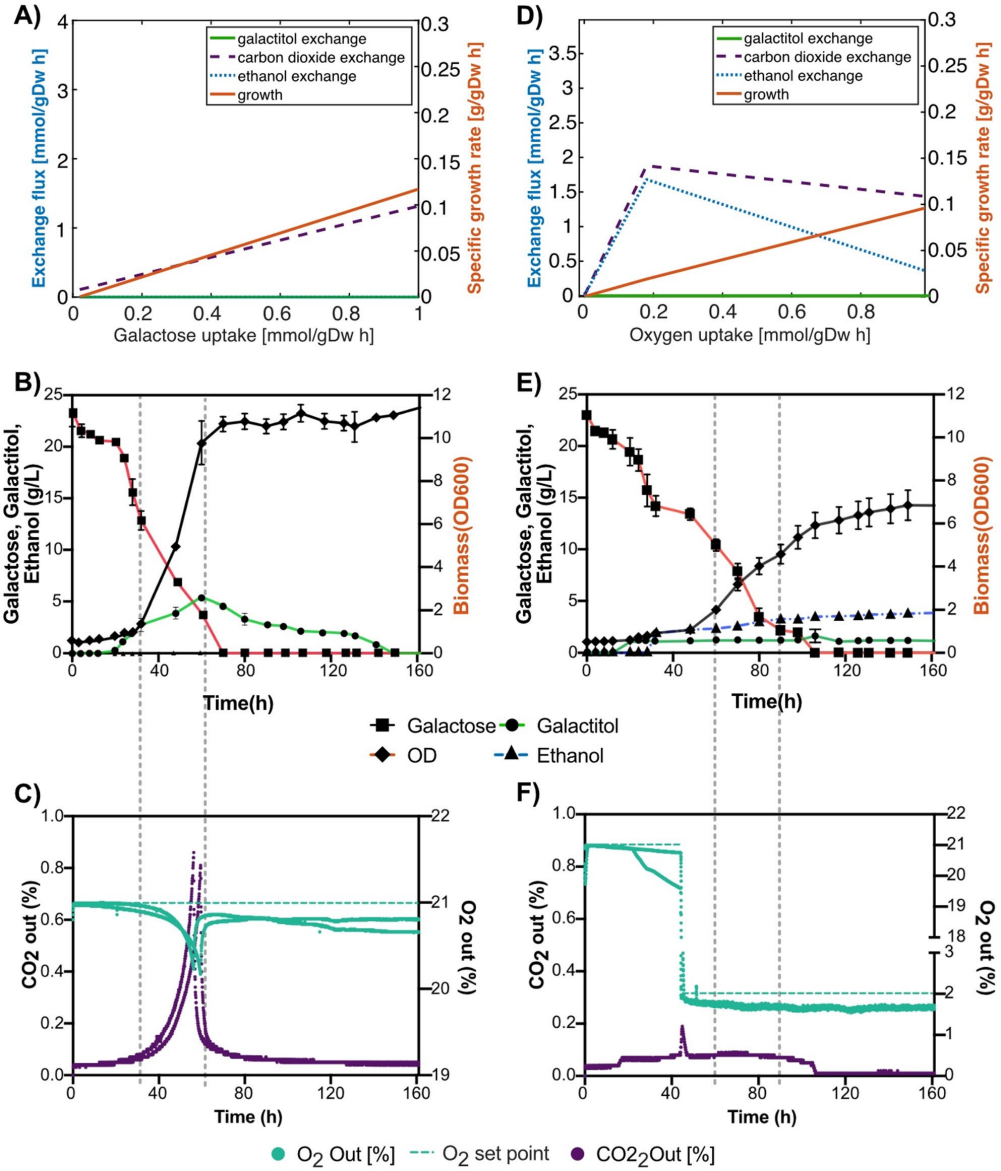
Model and quantitative fermentation unravel galactitol and ethanol secretion under nutrient-limited conditions. **A** Predicted growth rate and metabolites exchange at varying galactose uptake rates in *Sint-GEM*. Exchange flux (in mmol gDW^-1^ h^-1^) of galactitol (green solid), ethanol (dotted light blue), and CO_2_ (black dash line) shown on left y-axis, with specific growth rate (solid red line) on the right y-axis, are plotted against galactose uptake rate (in mmol gDW^-1^h^-1^). **B** Biomass accumulation and extracellular metabolite concentration over time for wild-type *S. intermedia* grown on batch reactors with standard aeration levels (21%). Biomass (OD, represented with diamonds), extracellular concentration of galactose (squares), galactitol (circles) and ethanol (triangles). **C** Percentage of O_2_ (aqua) and CO_2_ (purple) measured at the air outlet along the cultivation time in batch reactors with standard aeration levels (21% O_2_ as set point, indicated as an aqua-colored dotted line). Vertical grey dotted lines delimit the time span with the highest measured growth and galactose consumption rates. **D** Predicted growth rate and metabolites exchange at varying oxygen uptake rates in *Sint-GEM*. Exchange flux (in mmol gDW^-1^h^-1^) of galactitol (blue solid), ethanol (dotted light blue), and CO_2_ (black dash line) shown on left y-axis, with specific growth rate (solid red line) on the right y-axis, are plotted against oxygen uptake rate (in mmol gDW^-1^h^-1^). **E** Biomass accumulation and extracellular metabolite concentration over time for wild-type *S. intermedia* grown on batch reactors with low aeration levels (2% O_2_). Biomass (OD, represented with diamonds), extracellular concentration of galactose (squares), galactitol (circles) and ethanol (triangles). **F** Percentage of O_2_ (aqua) and CO_2_ (purple) measured at the air outlet along the cultivation time in batch reactors with low aeration levels. Vertical grey dotted lines delimit the time span with the highest measured growth and galactose consumption rates

FBA simulations under galactose-limiting conditions predicted complete respiratory metabolism without byproduct secretion (Table 1; Fig. 6A), a pattern also reflected in the PCA, which showed a tightly constrained solution space reflecting efficient carbon utilization without ethanol or galactitol secretion (Fig. S5A). In contrast, batch cultivation on galactose revealed significant levels of galactitol production (Fig. 6B). Reactor off-gas and metabolite analysis revealed that the time interval of the highest rate of galactitol production coincided with maximum cellular growth, the highest rate of galactose consumption and the highest (but non-depleting) oxygen uptake rates (between 36 and 60 h, marked by dotted lines in Fig. 6B, C). During extended cultivation, the secreted galactitol started to be consumed as galactose levels neared depletion in the media (60 h, Fig. 6B). This shift from net production to net consumption suggests that cells may be reaching primary carbon-limiting conditions and avoid any byproduct secretion, aligning with the predictions shown in Fig. 6A and the random sampling results under galactose-limited conditions (Table 1).

The observed aerobic galactitol production on galactose, contrasted with its absence in *Sint-GEM* FBA predictions under galactose-limited conditions, suggests that galactitol secretion may function as an overflow metabolite resulting from incomplete respiration of incoming galactose. Additionally, regenerated NAD^+^ coupled to galactitol overflow may be consumed by a high glycolytic flux, analogous to NAD^+^ regeneration by ethanol formation observed in *S. cerevisiae* [53]. This hypothesis could be thoroughly explored with the construction of an enzyme-constrained version of *Sint-GEM*, as this has been a useful modelling approach to shed light on overflow metabolism in other organisms across kingdoms of life [54–57].

Additionally, FBA simulations under galactose and lactose-limited conditions showed that a small fraction of the simulations predicted D-sorbitol and L-sorbose production, and L-sorbose was in fact detected in trace amounts in galactose containing media (Fig. S6). These observations support the predictions from random sampling and show that although secretion of these byproducts may be rare, it does happen (Table 1).

**Table 1:** Percentage of simulations secreting ethanol or oxidoreductive pathway intermediates in 10,000 FBA random-sampling simulations of *Sint-GEM* under different carbon and nutrient limitations (C = carbon, O₂ = oxygen, N = nitrogen)

Metabolite	Galactose			Lactose		
	C-limited	O_2_-limited	*N*-limited	C-limited	O_2_-limited	*N*-limited
Ethanol	0.13%	94.84%	0%	0.24%	100%	0
Galactitol	0.8%	60.51%	0.33%	0.11%	100%	42.93%
D-sorbitol	0.04%	0%	0%	0.09%	0%	0
L-sorbose	0.03%	0%	0%	0.13%	0%	0

### Oxygen-limiting conditions

Both random sampling and FBA predictions of the *Sint-GEM* solution space suggested that galactitol production is a prominent metabolic feature under both aerobic and oxygen-limited conditions. Nearly 60% of the galactose simulations under oxygen limitation displayed combined galactitol and ethanol secretion, reflected in the PCA as distinct yet partially overlapping metabolic regimes associated with these two byproducts (Fig. S5C). This phenotype was consistent with the experimental reactor data (Fig. 6D, E, F). Moreover, secretion of galactitol was also observed in aerobic cultivations (Fig. 6B, E), suggesting that its production is not dictated by oxygen availability. In contrast, secretion of ethanol seems tightly coupled to oxygen-limited cellular growth on galactose and lactose, occurring in over 94% of the predicted cases (Table 1).

Ethanol production under low-oxygen conditions is a common trait among budding yeasts that facilitates NAD⁺ regeneration, and *Sint-GEM* predicted this effect to be even more pronounced during growth on lactose, occurring in 100% of simulations (Table 1). A detailed exploration of flux distributions indicated that, in these scenarios, ethanol production serves to balance the NADH generated from the glycolytic oxidation of the glucose moiety in lactose. Parallelly, galactitol secretion can balance the additional NADH that is generated from the galactose moiety, mostly catabolized by the Leloir pathway down to glucose-1-phosphate and further integrated into glycolysis (Suppl. Table 6).

### Nitrogen-limiting conditions

Nitrogen-limited simulations in *Sint-GEM* predicted a strong shift toward galactitol secretion, particularly during growth on lactose (Table 1). This was reflected in the PCA, where nitrogen limitation resulted in frequent galactitol-secreting phenotypes that clustered along axes associated with Leloir and oxidoreductive pathway turnover, while ethanol secretion remained negligible (Fig. S5E–F). Constraining *Sint-GEM* with experimental growth, galactose uptake, and galactitol secretion rates revealed lower PPP flux and CO₂ release under nitrogen limitation than under oxygen limitation (Table S7), consistent with higher galactitol production due to reduced carbon flux redirection to the PPP for NADPH regeneration. Consistent with these predictions, bioreactor experiments showed that reducing ammonium sulphate from 5 g/L to 0.05 g/L increased galactitol yields by approximately threefold, despite reduced growth (Fig. S7), demonstrating that limiting nitrogen availability directs excess carbon toward galactitol formation.

Overall, the results on quantitative fermentation, supported by model predictions, highlight galactitol as a key overflow metabolite whose secretion is influenced by both oxygen and nitrogen availability. Extracellular accumulation of galactitol and ethanol together was more probable under oxygen limitations, consistent with redox balancing through multiple secretion pathways. In contrast, galactitol secretion alone occurred during aerobic growth on galactose, indicating that its formation can serve as a distinct overflow mechanism independent of oxygen limitation. Furthermore, galactitol secretion yields increased under nitrogen-limited conditions, suggesting a broader role in maintaining cellular redox balance during nutrient stress. Notably, these results also show that galactitol accumulation is not an exclusive trait of the *galΔ* mutant but also takes place in the wild-type strain.

### *Sungouiella intermedia* demonstrates significant cell factory potential for production of galactitol and tagatose

As galactitol is the precursor for the rare sugar tagatose and can be produced using microbial cell factories, the galactitol-overproducing *S. intermedia galΔ* strain represents a particularly suitable host. Thus, we aimed to leverage insights from *Sint*-GEM to optimize the bioprocess for galactitol production from lactose.

To identify growth conditions that maximize galactitol production and secretion, we first performed flux sampling of the *Sint-GEM* model on lactose for the *galΔ* strain. This also allowed us to test whether the metabolic trends observed for the wild-type strain on galactose extend to the mutant grown on lactose. Simulations indicated that galactitol is among the predominant secreted byproducts, occurring under both O_2_ and N-limited conditions, similar to the wild-type strain (Table S8. Notably, co-secretion of galactitol and ethanol was predicted in most cases, and more pronounced under oxygen-limited conditions. For experimental testing of these predictions, we grew the *galΔ* strain in minimal medium with 20 g/L lactose in both aerobic (ca. 21%) and low-oxygen (2%) conditions in batch reactors. Extracellular galactitol was detected in both conditions (4.8 and 1.2 g/L, respectively) and accompanied by ethanol (1.1 g/L) in the low-oxygen case (Fig. S8).

**Fig. 7:**
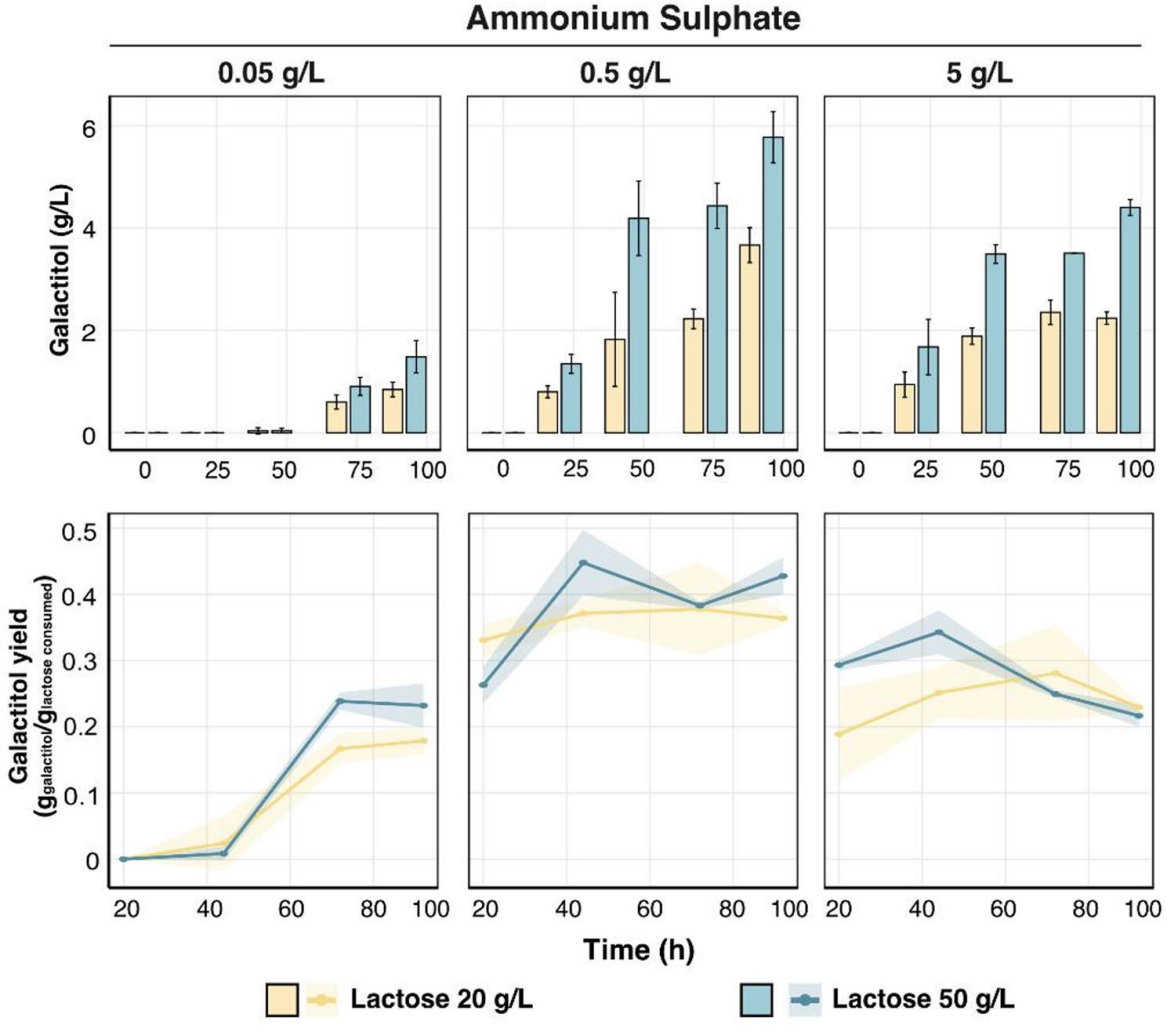
Effect of nitrogen concentration on galactitol titers and yields. Bar graphs showing galactitol titers (g per liter) and yields on lactose (gram of galactitol per gram of lactose consumed, plotted on Y-axis) against time (in hours) on X-axis during cultivation in minimal media. Each facet shows different Ammonium Sulphate concentrations (0.05, 0.5 and 5 g/L) tested in minimal media with either 20 or 50 g/L of lactose. Error bars represent standard deviation based on biological triplicates and statistical significance is determined using student’s t-test and is represented as “*” for *P* ≤ .05, “**” for *P* ≤ .01, and “***” for *P* ≤ .001

Under nitrogen-limited conditions, *Sint-GEM* predicted that galactitol secretion is favored in up to 99% of the cases in the *galΔ* strain (Table S8). To experimentally test this prediction, we varied the nitrogen concentration (0.05, 0.5, or 5 g/L ammonium sulfate) in minimal medium containing two different initial lactose levels (20 and 50 g/L). As observed for the wild-type strain, nitrogen availability influenced galactitol production in the *galΔ* mutant, and the highest galactitol titers and yields were achieved at 0.5 g/L ammonium sulfate for both lactose concentrations. At this nitrogen level, cultures grown with 20 g/L lactose produced 3.67 g/L galactitol (and 0.36 g galactitol per g consumed lactose), while those grown with 50 g/L lactose produced 5.78 g/L galactitol (and 0.45 g/g) (Fig. 7). Together, these results provide important guidance for choosing an oxygen-rich and nitrogen-limited bioprocess design when applying *S. intermedia galΔ* as a production host for conversion of lactose into galactitol.

**Fig. 8:**
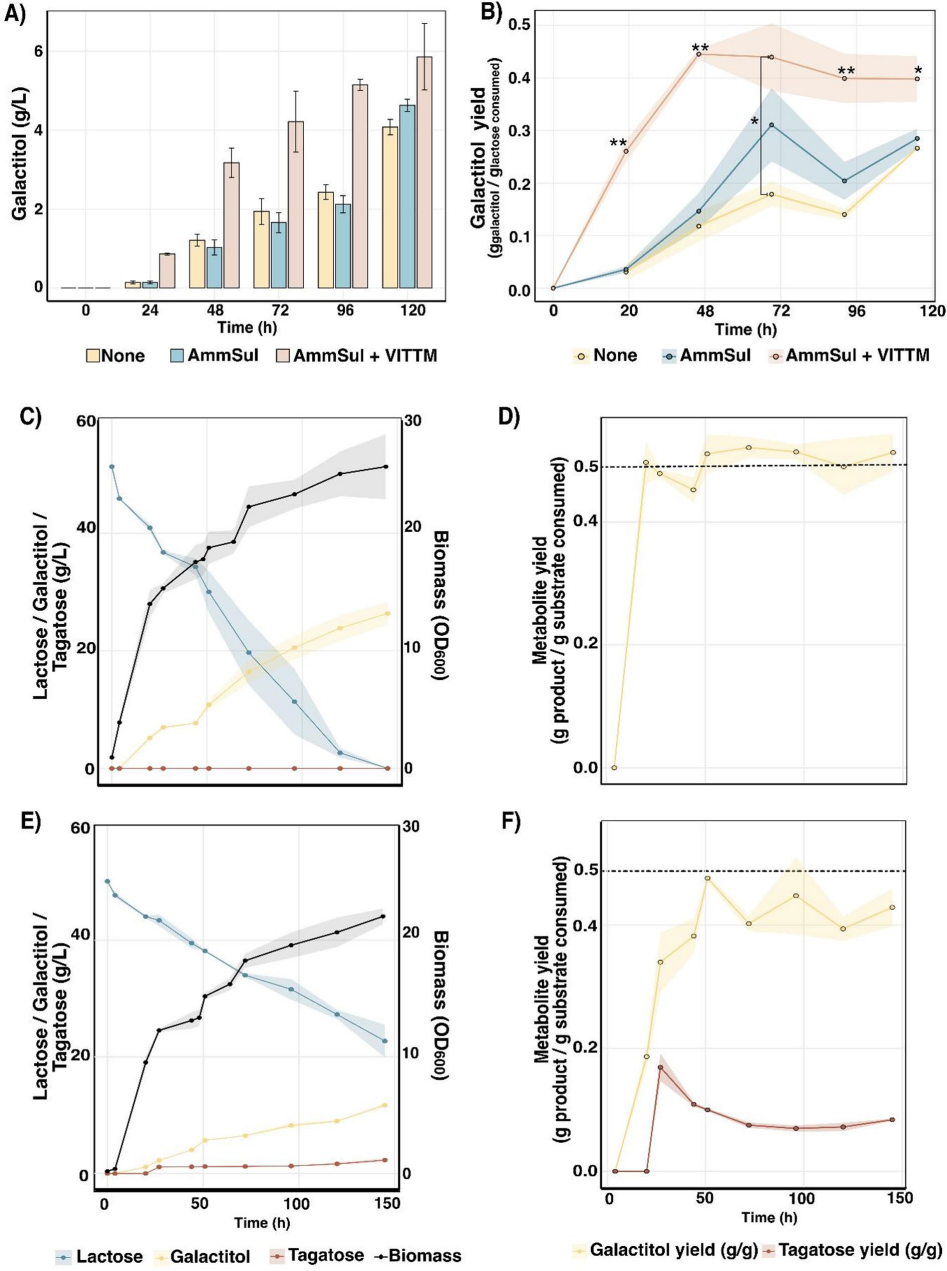
Effect of additional Ammonium Sulphate and Vitamins/trace metals to CWP on galactitol production (titers and yields) and Cell factory application of *S. intermedia galΔ* mutant for galactitol and tagatose production: **A** Bar plot showing galactitol concentration in g/L plotted on y-axis against time (in hours) for three culturing schemes: CWP without any additional ammonium sulphate (None - yellow), CWP with Ammonium Sulphate (AmmSul - Blue) and CWP with Ammonium sulphate and Vitamin/Trace metals (AmmSul + VITTM - Orange). **B** Line plot depicting changes in galactitol yield (calculated as gram of galactitol produced per gram of lactose consumed) plotted on y-axis against time (in hours) on x-axis for the three above mentioned culture schemes: None—yellow, AmmSul—Blue, AmmSul + VITTM—Orange. Error bars represent standard deviation based on biological triplicates and statistical significance is determined using student’s t-test and is represented as “*” for *P* ≤ .05, “**” for *P* ≤ .01, and “***” for *P* ≤ .001. **C** Line plot depicting galactitol production (yellow), lactose consumption (blue) in g/L plotted on left and biomass (black) on right y-axis against time (in hours). **D** Line plot depicting galactitol yield (gram of galactitol produced per gram of lactose consumed) on y-axis against time (in hours) on x-axis. The dotted line at y = 0.52 g/g represents theoretical maximum yield of galactitol from lactose. Error bars represent standard deviation for three biological replicates. **E** Line plot depicting tagatose (brown), galactitol production (yellow) and lactose consumption (blue) in g/L for *S. intermedia galΔ* equipped with a galactitol-2-dehydrogenase enzyme plotted on left and biomass (black) on right y-axis against time (in hours) on x-axis. **F** Line plot depicting tagatose and galactitol yield (gram of galactitol produced per gram of lactose consumed) on y-axis against time (in hours) on x-axis. The dotted line at y = 0.52 g/g represents theoretical maximum yield of galactitol from lactose. Error bars represent standard deviation for three biological replicates

To evaluate the suitability of the *S. intermedia galΔ* strain for converting an industrial feedstock into galactitol, we cultivated the strain in cheese whey permeate (CWP) containing 50 g/L lactose in aerated 1 ml culture plates in the Biolector. Since nitrogen availability, as well as vitamins and trace metals, likely influence both microbial growth and galactitol production, and their concentrations in CWP are not well defined, we supplemented the medium with these components and compared the strain’s performance. Addition of 0.5 g/L ammonium sulfate together with vitamins and trace metals (at added concentrations similar to those in the minimal medium) significantly improved the final galactitol titers (5.96 g/L) and cumulative yields (0.4 g/g) compared to CWP alone (4.1 g/L, 0.28 g/g) and CWP supplemented with ammonium sulphate only (4.5 g/L, 0.3 g/g) (Fig. 8A, B), showcasing the importance of addition of these nutrients for better cell factory performance.

Following the small-scale Biolector experiment, we assessed galactitol production capability of the *galΔ* mutant in 1 L bioreactors containing CWP and additional 0.5 g/L ammonium sulphate, and vitamins and trace metals. With a starting lactose concentration of 5%, the strain attained 26 ± 0.5 g/L galactitol as the highest titer after 150 h (Fig. 8C), maintaining a high yield of approx. 0.5 g galactitol produced per g lactose consumed (close to the theoretical maximum) during the whole fermentation (Fig. 8D), and reaching a productivity of 0.173 g/L^−1^h^− 1^.

Finally, we equipped the *S. intermedia galΔ* strain with a galactitol-2-dehydrogenase enzyme, enabling conversion of galactitol to the natural sweetener tagatose. Upon cultivation in CWP with ammonium sulphate and vitamins and trace metals, the engineered strain converted parts of the produced galactitol into tagatose, resulting in extracellular accumulation of 12 ± 0.6 g/L galactitol and 2.6 ± 0.2 g/L tagatose (Fig. 8E). Although the galactitol yield remained high (84% of theoretical maximum), this was not the case for the tagatose produced (~ 0.1 g tagatose produced per g lactose consumed, approx. 20% of the theoretical maximum) (Fig. 8F). The relatively low tagatose titers and yields indicate that the activity of the galactitol-2-dehydrogenase enzyme requires further optimization to achieve more efficient conversion of galactitol to tagatose. Moreover, the galactitol-2-dehydrogenase-expressing strain grew more slowly than the parental strain (Fig. 8C and E, and CO_2_ production is depicted in Fig. S9), likely reflecting a fitness cost from heterologous enzyme expression, possibly due to metabolic burden or cofactor imbalance. Future improvements could include optimizing enzyme expression and cofactor balance to enhance tagatose conversion and reduce metabolic bottlenecks. Nonetheless, our results demonstrate the applicability of *S. intermedia* strains as a potential cell factory for production of added-value products using CWP as feedstock.

## Conclusions

In this work, we demonstrated that *S. intermedia* possesses an oxidoreductive pathway operating in parallel with the Leloir pathway for galactose catabolism and highlighted its potential as a cell factory.

To this end, we curated a draft genome-scale model using bioreactor data (exchange fluxes, biomass composition), refined with orthology searches and RNA-seq data, and generated *Sint-GEM*: a high-quality model for simulating the metabolic landscape of *S. intermedia*. Flux balance analyses revealed that Leloir pathway plays a dominant role in lactose and galactose metabolism, while the oxidoreductive pathway is important for adaptation to various nutrient (carbon, oxygen, and nitrogen) limitations. Our findings further suggest that this secondary pathway plays a key role in dissipating excess carbon and supports overflow metabolism. Consistent with these predictions, both simulations and experiments revealed condition-dependent galactitol secretion and its subsequent reassimilation under carbon scarcity. Finally, by integrating *Sint-GEM* insights with experimental analysis, we demonstrated the cell factory potential of *S. intermedia*, optimizing galactitol production and enabling tagatose formation through heterologous pathway engineering.

Overall, this work establishes a framework for exploring and exploiting the biotechnological potential of the non-model yeast *S. intermedia* by combining genome-scale modeling with experimental systems biology. In the long term, applying this framework to other emerging yeast species may enable the development of scalable, high-performance bioprocesses that diversify and strengthen the industrial bioeconomy.

## Materials and methods

### *Sint-GEM* structure

*Sint-GEM* constitutes a catalogue of all enzyme-encoding genes, reactions and metabolites predicted to be part of *S. intermedia* metabolism. Metabolic reactions are represented by a stoichiometric matrix (***S***), which represents reactions in its columns and metabolites in its rows. Coefficients in this matrix represent the stoichiometric coefficient of a metabolite in each reaction it participates. As convention, negative coefficients are used for reaction substrates, while positive values represent reaction products. A two reactions example would take the following form:

$$\:{r}_{1}:\:\:\alpha\:A+\beta\:B\:\to\:\gamma\:C;\:{r}_{2}:\:\:\delta\:C\:\to\:\epsilon\:D+\zeta\:E$$

$$\:S=\left[\begin{array}{cc}-\alpha\:&\:0\\\:\begin{array}{c}-\beta\:\\\:\gamma\:\\\:\begin{array}{c}0\\\:0\end{array}\end{array}&\:\begin{array}{c}0\\\:-\delta\:\\\:\begin{array}{c}\epsilon\:\\\:\zeta\:\end{array}\end{array}\end{array}\right]$$

Similarly, relations between reactions and genes are stored in a reaction-gene matrix (***RG***), in which rows represent reactions in the model and columns the genes. If a gene product (enzyme) catalyzes a given reaction, then the corresponding coefficient in the ***RG*** matrix is assigned a value of 1. Rows with multiple non-zero elements indicate reactions with isoenzymes (i.e., different enzymes with equivalent catalytic activity) or reactions catalyzed by enzyme complexes, columns with multiple non-zero coefficients indicate promiscuous enzymes (i.e., enzymes with catalytic activity for multiple substrates and reactions). To differentiate between enzyme complexes or reactions with isoenzymes, an additional field relating reactions to genes is added to the model, *grRules*, indicating the gene(s) encoding for all enzymes related to each reaction. Rules of the form $\:{g}_{i}\:or\:{g}_{j}$ indicate reactions with isoenzymes. Rules of the form $\:\left({g}_{i}\:and\:{g}_{j}\right)$ indicate reactions catalyzed by enzyme complexes, in this case composed by subunits *i* and *j*.

### Standardization of *Sint-GEM*

A draft model reconstruction, based on yeast8 consensus metabolic model for *S. cerevisiae* and orthology searches across 332 budding yeasts and 11 filamentous fungi annotated genomes from a previous study was downloaded from: https://github.com/SysBioChalmers/Yeast-Species-GEMs/blob/main/Reconstruction_script/ModelFiles/xml/Candida_intermedia.xml. Missing steps in the galactose oxidoreductive pathway were inferred from orthology searches between *S. intermedia* genome and those of *Aspergillus niger*,* Aspergillus nidulans* and *Trichoderma reesei* using OrthoFinder v.2.5.4. Missing transport steps for lactose and galactitol were added based on observed phenotypes.

Biomass composition was modified in the model in accordance with total protein and total lipids measurements from cultures of *S. intermedia* strain CBS 141442 (see below). Amino acid composition for the global protein synthesis pseudoreaction in the model was modified according to amino acid ratios in the protein sequence of *S. intermedia* strain CBS 141442. Growth-associated and non-growth associated maintenance ATP expenditure, as well as the P/O ratio in the electron transport chain were fitted using RMSE minimization in prediction of experimental values for glucose and oxygen uptakes, and carbon dioxide production from glucose-limited chemostats at 0.05 and 0.1 h^− 1^ dilution rate.

Curation, parameter fitting, and model standardization were tracked using the git version control system and adhering to standard-GEM guidelines^14^, thus facilitating reproducibility, traceability and further development of the resulting GEM^15^. All model files and curation scripts are publicly available at: https://github.com/SysBioChalmers/sint-GEM.

### FBA simulations for optimal growth

Cellular growth simulations under a given nutrient limitation were performed using the flux balance analysis method, which assumes steady state for the intracellular concentrations of metabolites, and phenotype prediction by optimization of a cellular objective, thus yielding the following linear programing problem:

$$\:max.\:or\:min.\:\:\boldsymbol{Z}={\boldsymbol{C}}^{\boldsymbol{T}}\boldsymbol{v}$$

Subject to

$$\:\boldsymbol{S}\:\cdot\:\boldsymbol{v}=0$$

$$\:\boldsymbol{l}\le\:\boldsymbol{v}\le\:\boldsymbol{u}$$

Where $\:\boldsymbol{Z}$ is the optimal value of the objective function; $\:{\boldsymbol{C}}^{\boldsymbol{T}}$ is a row vector of coefficients of the objective function for each element in $\:\boldsymbol{v}$; $\:\boldsymbol{v}$ is the vector of intracellular reaction fluxes, in units of mmol/gDw h; $\:\boldsymbol{S}$ is the stoichiometric matrix described above; $\:\boldsymbol{l}$ and $\:\boldsymbol{u}$ are vectors of lower and upper bounds, respectively, for the reaction fluxes in $\:\boldsymbol{v}$. Cellular growth was simulated maximizing the biomass production pseudoreaction in *Sint-GEM* (r_4041), setting an arbitrarily low value of 1000 mmol/gDw h as lower bound for the exchange reactions of components in minimal essential media, and a decreasing value from 0 to -1 mmol/gDw h as lower bounds for the galactose/lactose and oxygen exchange reactions, depending on the simulated limiting condition. All exchange reactions were constrained by an upper bound of 1000 mmol/gDw h to allow secretion of metabolites. The RAVEN toolbox v2.8.7was used for all FBA simulations, with Gurobi v.10 as numerical solver and MATLAB R2022b.

### Random sampling of *Sint-GEM* solution space

A series of 10,000 FBA simulations with randomly generated coefficient vector ($\:{C}^{T}$) for the objective function for carbon-, oxygen- and nitrogen-limited conditions under lactose and galactose as sole carbon sources. For each case an initial growth maximization with a unit carbon source uptake rate as a constraint was simulated, as described above, to obtain $\:{v}_{growth}^{*}$ which is then used to set a lower bound in the growth pseudoreaction as $\:0.5*{v}_{growth}^{*}$ (suboptimal growth rate). A lower bound of -1 mmol/gDw h is set as lower bound for the exchange reactions of galactose, lactose exchange in the carbon-limited cases, oxygen exchange for oxygen-limited condition, and ammonium for the nitrogen-limited cases. When carbon is not the limiting element, then the lower bound for the galactose or lactose exchange reaction was set to -1000 mmol/gDw h. Minimal media components uptake and exchange metabolites secretion was allowed as described above. All random sampling simulations were run with the *randomSampling.m* function in the RAVEN toolbox.

### Analysis of metabolite turnover distributions

All flux distributions simulated by random sampling were converted to metabolite turnover distributions, in which for each metabolite turnover ($\:{\tau\:}_{i}$)

$$\:{\tau\:}_{i}=\frac{1}{2}\sum\:_{j}\left|{S}_{ij}{v}_{j}\right|$$

This quantity represents the total rate of transformation of metabolite *i* across all reactions in which it partakes (as substrate or product). For each simulated limiting condition, a matrix containing each of the computed metabolite turnover distributions for the respective 10,000 simulations as columns. Dimensionality reduction by principal component analysis (PCA) was performed on such matrix for each of the simulated conditions using the *prcomp* function in R v4.4.1. The top metabolites contributing to the mapping of samples (metabolite turnover distributions of all simulations) onto the first two principal components were obtained by identifying all metabolites with a weight coefficient on the same order of magnitude as the maximum coefficient for each PC (the minimum for the negative directions in PCs).

### RNA seq analysis and gene expression correlation analysis

Transcriptomics data from RNA sequencing was adopted from previous work, and the RNA seq datasets are available in the European Nucleotide Archive (ENA) with the accession number E-MTAB-6670 [14, 17].

For all genes included in *Sint-GEM*, normalized RNA expression values across conditions (glucose, lactose, galactose, cellobiose and xylose as sole carbon sources) were obtained from the RNAseq dataset. Pearson correlation coefficient and t-student statistical test was computed for all possible pairs of gene expression vectors using the *corrcoef* function in MATLAB R2022B.

### Determination of biomass composition

To determine biomass composition, *S. intermedia* was cultivated in a carbon-limited chemostat in biological triplicates. An overnight culture was prepared in minimal Verduyn medium [58] (hereafter referred to as minimal medium) containing 2% glucose or lactose. It was then inoculated into controlled stirred 1 L bioreactor vessels (DASGIP, Eppendorf, Hamburg, Germany) containing 500 mL minimal media with 2% glucose or lactose, respectively. Reactor conditions were maintained as: Temp = 30 °C; pH = 5.5 (maintained with 2 M Potassium Hydroxide); Aeration = 1 Vessel Volume per Minute; stirring = 300 rpm. Cultures were allowed to grow until stationary phase before turning on dilution of 0.1 h^− 1^ and 0.05 h^− 1^. After the cultures had reached steady state (as determined by CO_2_out and cell dry weight measured at an interval of at least 12 h), cells were harvested for measurement cell dry weight, total proteins and lipids as outlined below.

The original Yeast 8 model’s biomass reaction was used as the starting point for the biomass reaction in draft *Sint-GEM*. Experimentally determined specifications from *S. cerevisiae* already present in *Sint-GEM* were taken; specifically inorganic compounds (i.e., phosphate, sulfate, and metal ions), and cell wall compositions, as corresponding data for *S. intermedia* CBS 141442 were not available. Additional data adopted from the yeast 8 model were Genome GC content, relative abundance of acyl groups and free fatty acids in lipids, lipid subspecies composition (e.g., phosphatidylinositol, phosphatidylcholine, phosphatidylethanolamine, and phosphatidylserine composition). In addition, we experimentally determined *S. intermedia*’s total proteins and lipids, to fine-tune the metabolite coefficients in the biomass reaction in *Sint-GEM*. Moreover, metabolite coefficients associated with growth-associated ATP maintenance were updated in *Sint-GEM* using experimentally determined rates from bioreactor cultures (Suppl. Table 5).

### Total proteins

Protein was measured using the Lowry method. Briefly, 20 mL culture was pelleted by centrifugation at 10,000 x g for 5 min, washed and resuspended in 10 ml. An aliquot of 2 ml from this is mixed with 1 ml of 3 M NaOH. Cell suspension was incubated at 100 °C for exactly 10 min and then immediately cooled on ice. An aliquot of 0.45 ml of the sample was taken in a clean tube, to which 0.45 ml 1 M NaOH and 0.3 ml of Copper Sulphate (2.5% w/v) were added. The solution was mixed well and incubated at room temperature for 5 min, following which, the mix was centrifuged at 10,000 x g for 5 min. Absorbance was measured of the clear supernatant at 510 nm. For calibration curve, Bovine Serum Albumin (Thermo), at a starting concentration of 5 mg/ml was used. Analysis was performed with biological triplicate samples derived from carbon-limited chemostats.

### Total lipids

Dry biomass was weighed in glass tubes (100 mg) and resuspended in Chlorophorm-Methanol (2:1) solution and heated to 80 °C. Tubes were flushed with nitrogen and dried overnight to measure the total lipid in each sample. Finally, tubes were weighed to determine the total lipid content per g of biomass. Analysis was performed with biological triplicate samples derived from carbon-limited chemostats.

### Molecular techniques, cloning and strain engineering

#### Cloning and *E. coli* transformation

Gene deletion mutants of *S. intermedia* (*lxr4*, *lad1*, *xyl2*) were constructed using the split-marker technique developed for this yeast, as described in detail in [16], and previously applied to deletion of the *GAL* cluster [17]. Briefly, deletion cassettes consisting of ~ 1 kb upstream and downstream homology arms fused to complementary halves of the *CaNAT1* selection marker were assembled by Gibson assembly following the NEBuilder protocol (New England Biolabs, USA). Each half-cassette was then PCR-amplified to generate the split-marker fragments used for transformation.

Similarly, the galactitol-2-dehydrogenase expression cassette was constructed in two parts, using Golden Gate cloning. The first part consisted of ~ 1000 bp upstream homology to *SiADE2*, the *SiTEF1* promoter driving the *Rl*GDH gene (From *Rhizobium leguminosarium*) with *SiACT1* terminator and one half of the *SiADE2* gene, and the second part consisted of the remaining half of *SiADE2* and ~ 1000 bp downstream homology to *SiADE2*. Both parts were cloned using the BsmBI cloning site of the plasmid *pGGAselect* ordered from Addgene (https://www.addgene.org/195714/) [20, 59], and PCR was used to amplify the integration fragments before transformation.

For amplification of plasmids, *E. coli* DH5α cells were transformed using heat-shock method [60], followed by selection on agar plates containing LB medium (1% tryptone, 1% NaCl and 0.5% yeast extract) and chloramphenicol (25 µg/mL) or ampicillin (100 µg/mL). Transformants were subsequently grown in liquid LB medium supplemented with the appropriate antibiotic for plasmid amplification, and plasmids were thereafter purified using GeneJET Plasmid Miniprep Kit (ThermoFisher, USA) as per manufacturer’s instructions.

#### *S. intermedia* strain engineering

Transformation of split-marker fragments into the *S. intermedia* wild-type strain was carried out as previously described [17]. Briefly, overnight cultures were harvested and washed twice with ice-cold Milli-Q water (MilliporeSigma, USA). Cells were resuspended in cold 1 M sorbitol and recentrifuged, after which fresh 1 M sorbitol was added to prepare competent cells at a ratio of 160 µL sorbitol per 1 OD of cells. The suspension was divided into 40 µL aliquots for transformation. To each aliquot, 8 µL of DNA (approximately 2 µg) was added and the mixture transferred to a chilled 0.2-cm electroporation cuvette (BioRad, USA). Electroporation was performed at 1.5 kV using a BioRad MicroPulser, followed by immediate recovery in rich medium containing 1 M sorbitol at 30 °C for 3 h with gentle shaking (1 × g). Cells were then plated on rich medium supplemented with Nourseothricin (200 µg/mL) and incubated at 30 °C for up to 48 h until colonies appeared. Nourseothricin resistant transformants were screened for targeted deletions using colony PCR. Single colonies were picked, re-streaked to ensure pure strains, and transferred to 200 µl PCR tubes containing 20 µl milliQ using sterile toothpicks. The PCR tubes were placed in a thermocycler and heated to 90 °C for 10 min followed by subsequent cooling to 12 °C. For each colony picked, 2 µl of the heated colony material was used as template for PCR using PHIRE II polymerase. Screening for transformants using PCR was performed by employing primer pairs with one primer binding to the genome outside the homology flank of the integration fragment and one in the marker gene. A third primer binding to the target gene was used added to the mix, to identify transformants with incorrect integrations. The tagatose production strain was constructed in a *galΔade2Δ* strain background. The *galΔade2Δ* strain was able to grow slowly on minimal media agar plates containing 2% glucose and formed small, visibly red colonies. Integration of the *RlGDH* expression cassette in the *galΔade2Δ* strain was performed using the split-marker technique and electroporation, as described above, and cells were plated on minimal media agar devoid of adenine. White transformants indicated integration of the complete *ADE2* gene. These colonies were re-streaked, and integration of the whole *RlGDH* expression cassette was confirmed by PCR.

### Growth characterization

#### Cell growth quantifier (CGQ)

Growth characterization was performed in 100 ml shake flasks using the CGQ system (aquila biolabs GmbH, Germany) [61]. Wild-type and mutant strains were precultured at 30 °C, 200 rpm overnight in minimal medium containing 2% glucose (w/v) and thereafter inoculated in 25 ml of minimal medium supplemented with 2% lactose to a starting OD_600_ of 0.1. Growth was quantified as “Scatter values” by the aquila system-based on the scatter light detected by the sensors. All cultivation conditions were investigated in triplicates.

### Growth profiler

Wild-type and mutant strains of *S. intermedia* were cultivated in 96-well plate format using the Growth Profiler 960 (Enzyscreen, The Netherlands) to follow growth over time. Strains were precultured at 30 °C, 200 rpm overnight in minimal medium with 2% galactose as sole carbon source. Precultured cells were then inoculated in 200 µL minimal media supplemented with 1% galactitol to achieve starting OD_600_ = 0.25. ‘Green Values’ (GV) measured by the Growth Profiler 960 (EnzyScreen, The Netherlands) correspond to growth based on pixel counts, and GV changes were recorded every 30 min for 72 h at 30°C and 250 rpm. All cultivation conditions were investigated in duplicates or triplicates.

### Biolector

Yeast colonies from an agar plate were inoculated the day prior to the screening in 5 mL minimal medium containing 2% lactose and grown overnight at 30 °C in 50-mL tubes. Cells were washed twice in milliQ water (centrifuged at 3000 rpm) and then inoculated into minimal medium with different carbon sources or industrial cheese whey permeate (WheyCo Inc.) to a final volume of 1 ml using Biolector XT flower plates (Beckman Coultier, USA) and sealed with AeraSeal films (Sigma-Aldrich). Initial OD600 was set to 1. The temperature was set to 30 °C with 85% humidity, shaker frequency was 1300 rpm, and cycle time was 30 min. All cultivation conditions were investigated in triplicates.

### Bioreactors

In addition to characterization of the wild-type strain in carbon-limited chemostats, *S. intermedia* strains (wild-type (*S. intermedia* CBS141442) and mutant strains) were cultured in stirred 1 L bioreactors (DASGIP, Eppendorf, Germany) containing 500 mL of either minimal medium containing galactose/lactose or cheese whey permeate. Batch reactor runs were performed at 30 °C, pH = 5.5 (maintained with 2 M Potassium Hydroxide) and aeration coupled with stirring was set to cascade mode to maintain dissolved oxygen at 30% as lowest value. Cells were harvested for biomass measurement and metabolite analysis at regular intervals. All culture conditions were investigated in duplicates.

### Metabolite analysis using HPLC

Changes in metabolite (lactose, galactose, glucose, ethanol and galactitol) concentrations were quantified using Jasco high-performance liquid chromatography (HPLC) system (Jasco Corporation, Japan) equipped with an RID-10 A refractive index detector, a Rezex RCM Monosaccharide Ca(^+2^) (8%) column (Phenomenex Inc., USA) with MilliQ Water (Merck MilliPore, USA) as the mobile phase and eluted at a flow rate of 0.6 mL/min at 80 °C. For the detection of Tagatose and Sorbose, Dionex ICS-5000+ (ThermoFisher, USA) was applied using the monosaccharide separation column, Dionex™ CarboPac™ PA1 (ThermoFisher, USA). Separation was performed using MilliQ Water as the mobile phase and eluted at a flow rate of 1 mL/min at 70 °C. Prior to analysis, culture samples were pelleted, and the supernatant was passed through 0.22 μm polyethersulfone syringe filter. Chromatogram peaks were treated and integrated using the software ChromNav v2.0 (Jasco HPLC) or Chromeleon v6.8 (Dionex IC).

## Supplementary Information

Below is the link to the electronic supplementary material.

Supplementary Material 1

## Data Availability

All data is provided within the article and supplementary material or can otherwise be provided by the corresponding author upon reasonable request. The development of Sint-GEM was tracked and the details can be found at https://github.com/SysBioChalmers/sint-GEM. Transcriptomics data from RNA sequencing was adopted from previous work, and the RNA seq datasets are available in the European Nucleotide Archive (ENA) with the accession number E-MTAB-6670.
