# Supplementary material for: Model-driven exploration of lactose and galactose metabolism via an oxidoreductive pathway in Sungouiella intermedia for cell factory applications
Source: Microb Cell Fact. 2026 Feb 1;25:60. doi: 10.1186/s12934-026-02941-y (PMC12930983; doi:10.1186/s12934-026-02941-y)
Supplement: Supplementary file 1 — Supplementary Material 1 [file 12934_2026_2941_MOESM1_ESM.docx]

# Supplementary Material

Supplementary Tables 1-8 can be found here: <https://github.com/SysBioChalmers/sint-GEM>


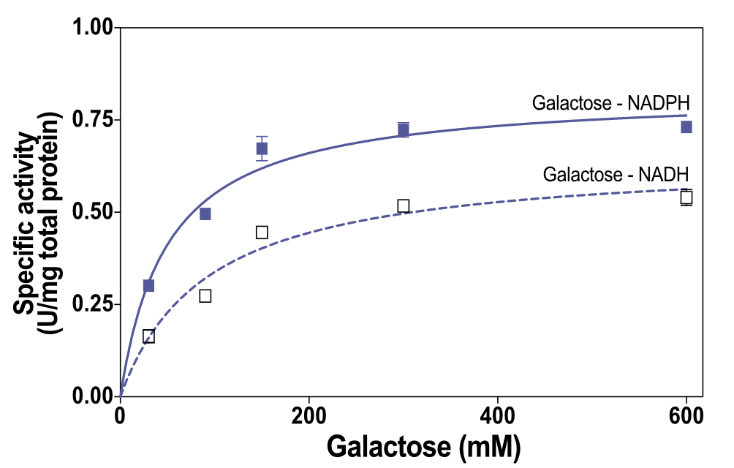


**Figure S1. Enzymatic activity for the determination of co-factor specificity of aldose reductase in *S. intermedia*.** Line plot depicting enzyme specific activity on y-axis as units/mg total protein against galactose concentration using NADH/NADPH as cofactors. Error bars represent standard deviation based on biological triplicates.


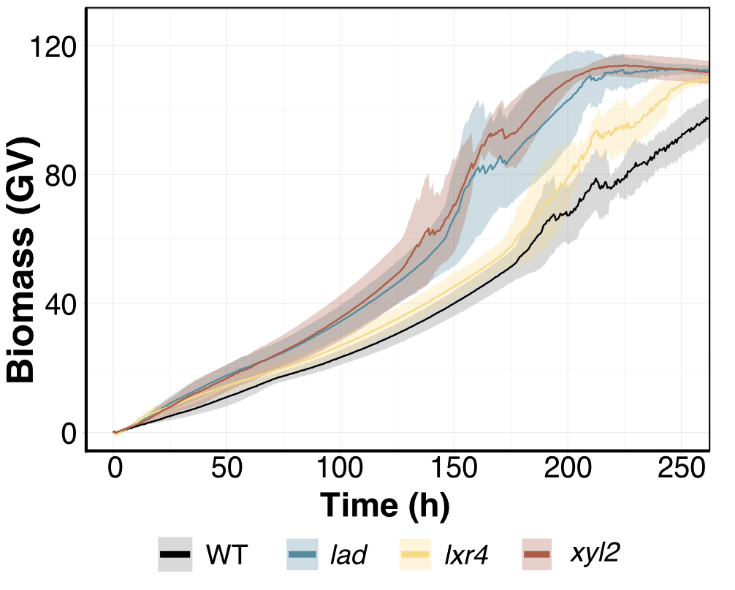


**Figure S2. Growth curves for wild-type and deletion mutants**. Wild-type (WT), and hypothesized oxidoreductive pathway mutants (ladΔ, lxr4Δ, xyl2Δ) in minimal media containing 1% galactitol. Graph indicates Time (h) on x-axis plotted against Biomass as Green Values (G.V.) as determined in a 96 well plate in the growth profiler equipment. Error is depicted as shaded area and represents standard deviation based on biological triplicates.


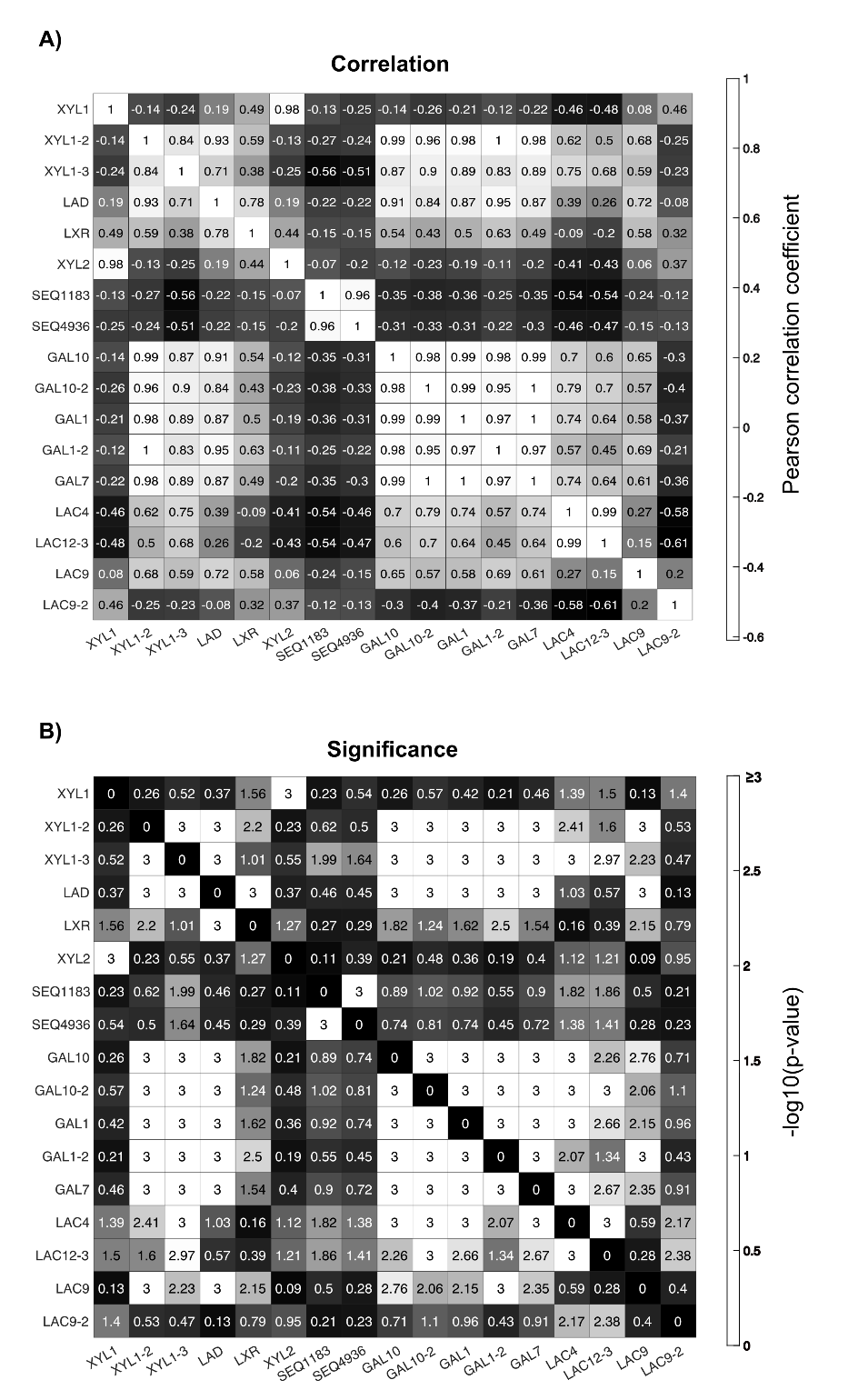


**Figure S3. Gene expression correlation analysis.** mRNA Expression levels across growth on 5 different carbon sources (glucose, xylose, cellobiose, galactose and lactose) for genes in the oxidoreductive and Leloir galactose metabolizing pathways were compared against each other. A) Pearson correlation coefficients for each pairwise comparison of gene expression vectors are shown with numbers and a grey color scale. B) Significance of linear correlation expressed as -log10(p-value) is shown in numbers and as a grey color scale. Hypothesis testing was performed using a two-tailed t-test statistic.


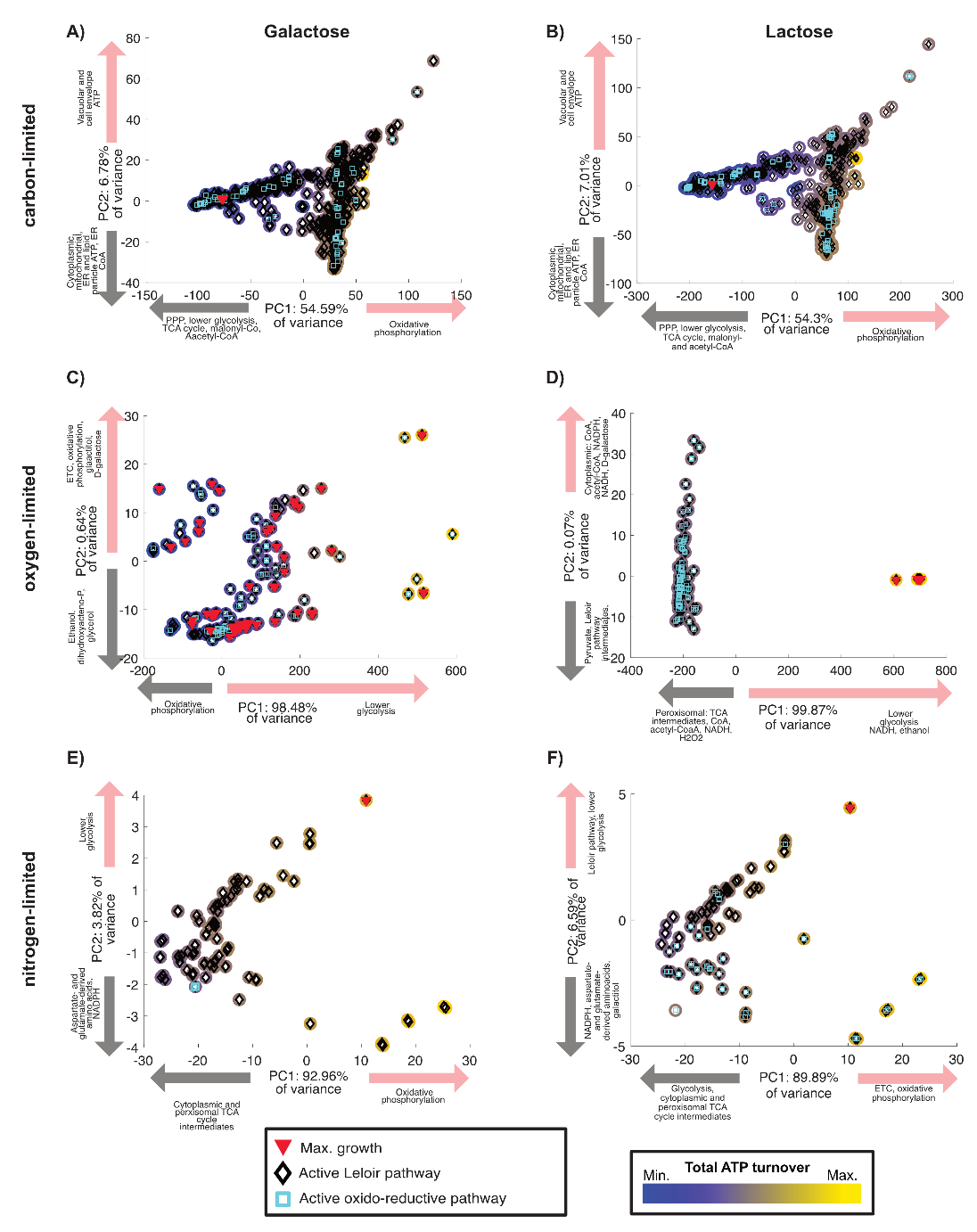


**Figure S4. PCA of galactose pathway utilization.** 10,000 randomly sampled flux distributions simulations are converted into metabolite turnover distributions for each case. Simulated conditions are growth on galactose under carbon-limitation (a), oxygen-limitation (c), nitrogen-limitation (e); and growth on lactose under carbon-limitation (b), oxygen-limitation (d), nitrogen-limitation (f). Global ATP turnover was mapped onto the color scale for each simulation point (dark blue for minimum ATP turnover – bright yellow for maximum values). Simulations displaying maximum growth rate under the specified constraints are marked with red filled triangles, those in which the Leloir pathway is carrying flux are shown with black non-filled diamonds, and those in which the galactose oxidoreductive pathway carries flux are highlighted with non-filled aqua squares. Metabolites and pathways with the highest contribution to positive and negative values of PC1 and PC2 are displayed along the X and Y axis, respectively.


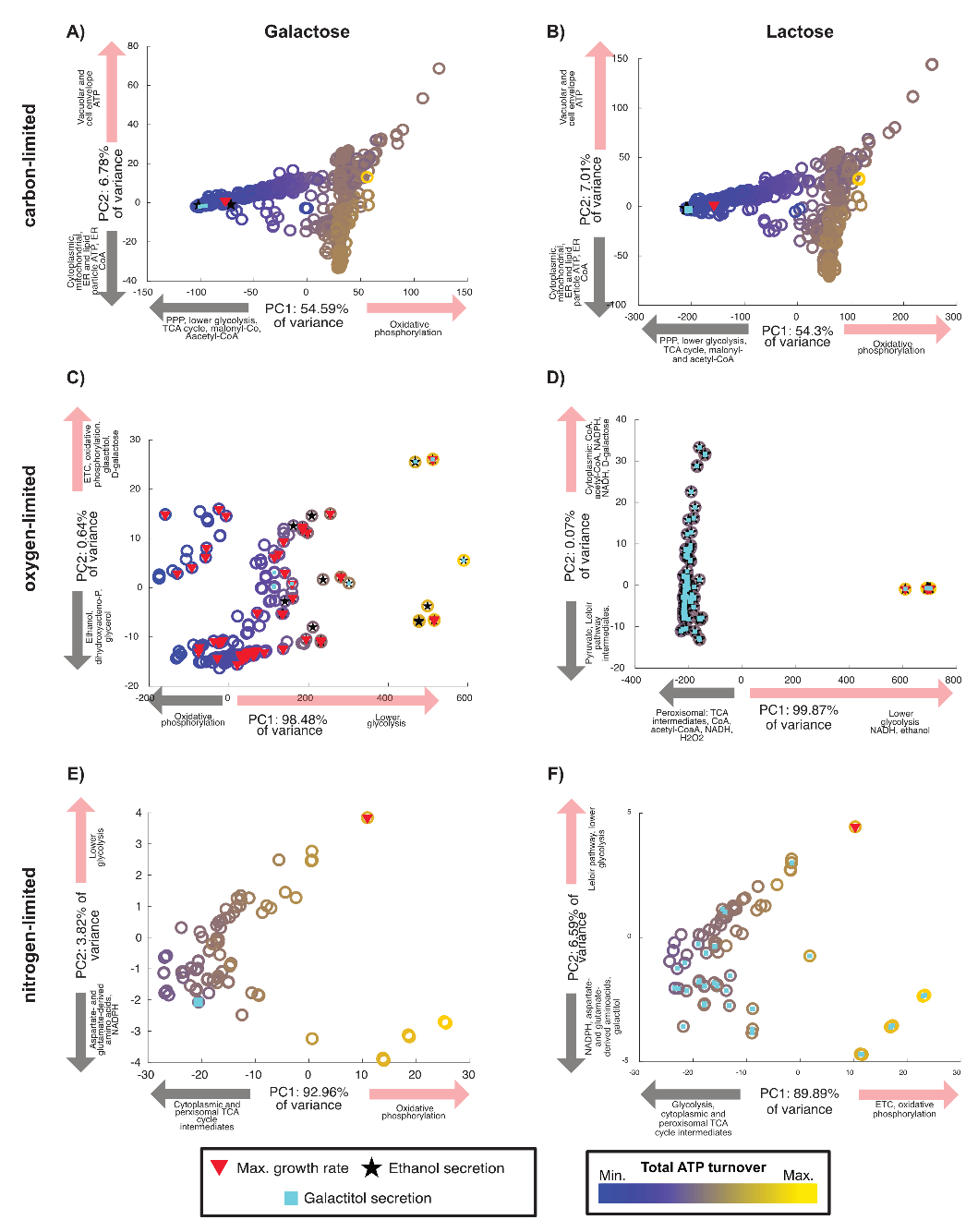


**Figure S5. PCA of byproduct secretion.** 10,000 randomly sampled flux distributions simulations are converted into metabolite turnover distributions for each case. Simulated conditions are growth on galactose under(A) carbon-limitation, (C) oxygen-limitation, (E) nitrogen-limitation; and growth on lactose under (B) carbon-limitation, (D) oxygen-limitation, (F) nitrogen-limitation. Global ATP turnover was mapped onto the color scale for each simulation point (dark blue for minimum ATP turnover – bright yellow for maximum values). Simulations displaying maximum growth rate under the specified constraints are marked with red triangles, those predicting ethanol secretion are mapped with black pentagrams, and those galactitol secreting simulations with aqua squares. Metabolites and pathways with the highest contribution to positive and negative values of PC1 and PC2 are displayed along the X and Y axis, respectively.


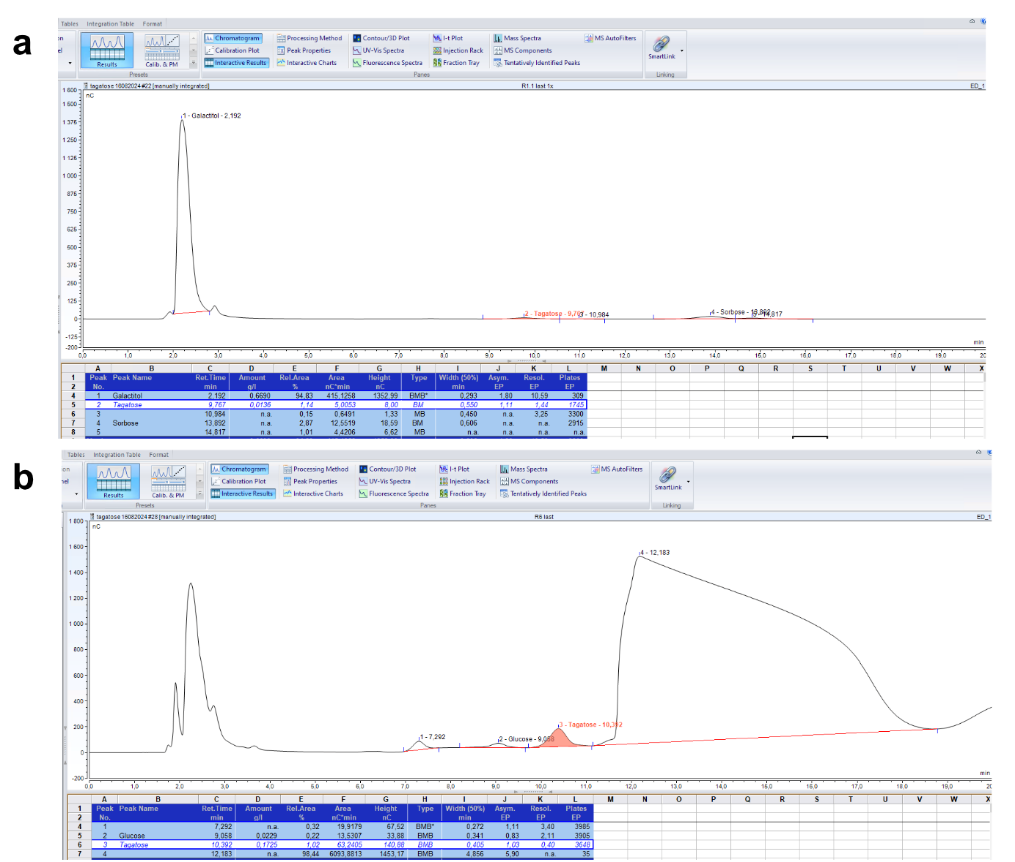


**Figure S6. Chromatogram from ion-chromatography analysis of samples from bioreactor run with the cultivation of *S. intermedia*** wild-type strain in minimal media containing 2% galactose as carbon source and 5% ammonium sulphate (High Nitrogen condition) in the media. Culture was maintained at pH 5.5 and with 21% oxygen in the inlet air. Galactose was completely consumed at the endpoint and L-sorbose was detected in addition to galactitol in the media. Compounds were detected against internal standards for the respective compounds.


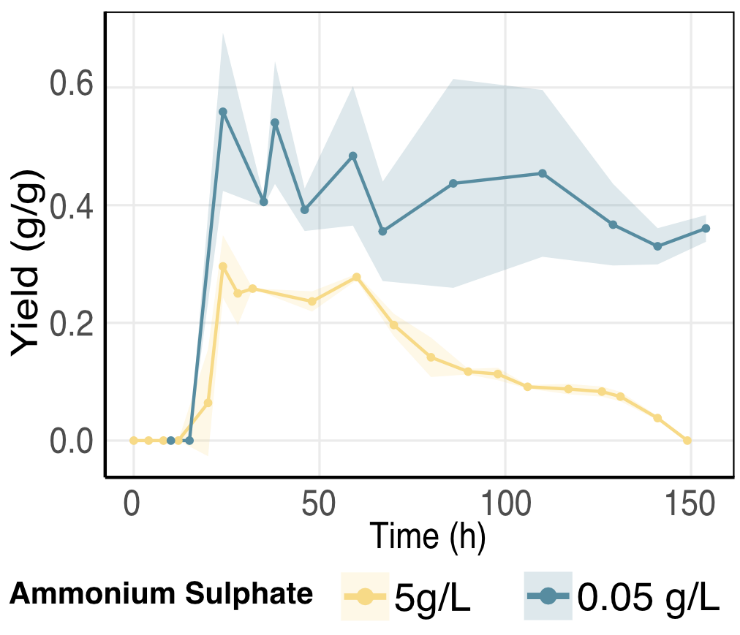


**Figure S7. Experimental validation of *Sint-GEM* results for flux sampling in nitrogen-limited conditions**. Graph depict the yields of galactitol and the biomass of the wild-type strain in minimal media containing two different concentrations of ammonium sulphate (0.5% = high and 0.005% = low). The graphs represent Yield in gGalactitol/gConsumedGalactose on the left y-axis and the biomass (OD600) formation on the right y-axis, plotted against Time(h) on the x-axis. Error is depicted by shaded area and represents standard deviation based on biological triplicates.


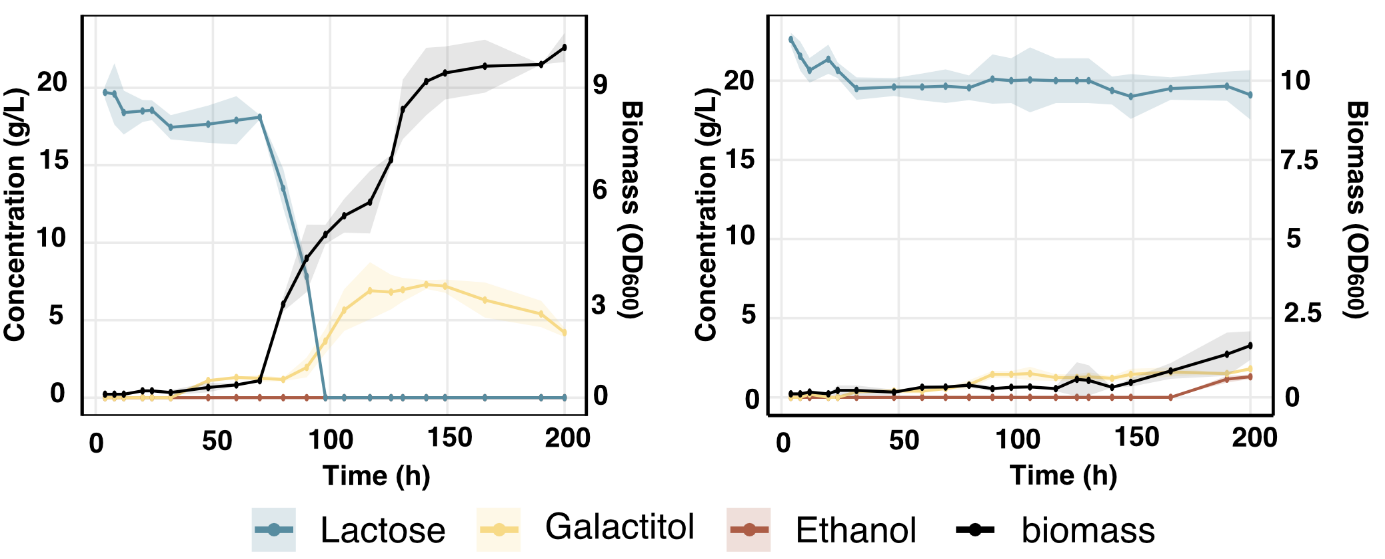


**Figure S8. Growth and metabolite production profiles from batch culture in 1L bioreactor for aerobic (21% oxygen in inlet air) vs low-oxygen (2% oxygen in inlet air):** Cultivation of gal strain minimal media with 2% lactose as starting substrate. Culture conditions were maintained as mentioned in the materials and methods section except for the percentage of oxygen supplied in the inlet air. Error *is depicted as shaded area* *and* represent*s* standard deviation based on biological triplicates*.*


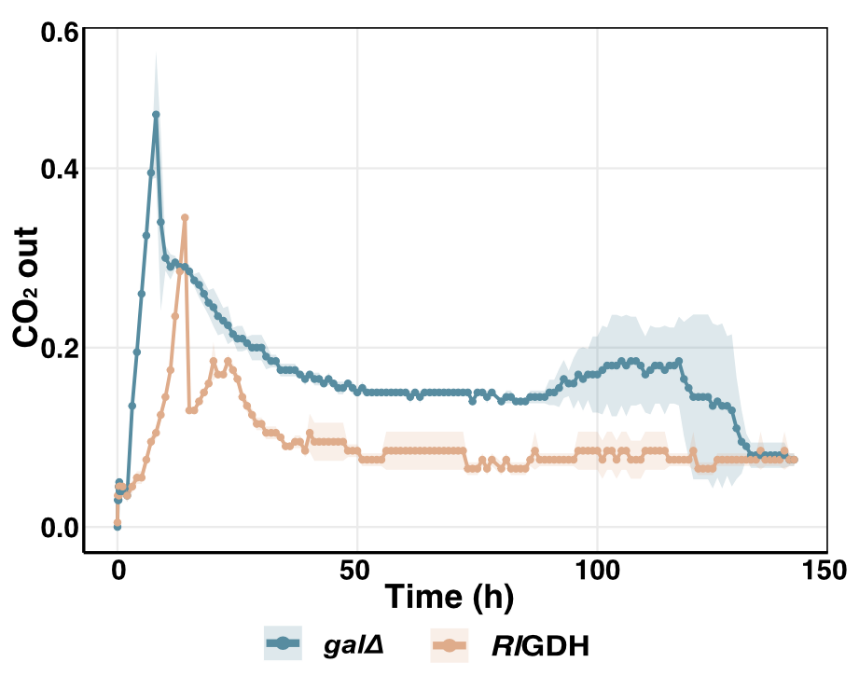


**Figure S9. Carbon dioxide profiles from batch culture in 1L bioreactor** Cultivation of gal strain minimal media with 2% lactose as starting substrate. Culture conditions were maintained as mentioned in the materials and methods section except for the percentage of oxygen supplied in the inlet air. Error *is depicted as shaded area* *and* represent*s* standard deviation based on biological triplicates*.*
